# Supplementary material for: 4D-DIA proteomics reveals distinct proteolytic landscapes induced by mechanical stress, Agrobacterium, and a viral capsid precursor
Source: PLoS One. 2026 Sep 11;21(9):e0356905. doi: 10.1371/journal.pone.0356905 (PMC13567786; doi:10.1371/journal.pone.0356905)
Supplement: Supplementary file 2 [file pone.0356905.s010.pdf]

Response to Reviewers — PONE-D-26-17969

Manuscript: 4D-DIA proteomics reveals distinct proteolytic landscapes induced by mechanical stress, *Agrobacterium*, and complex viral particles

We sincerely thank the Academic Editor and both Reviewers for their careful and constructive evaluation of our manuscript. The comments have substantially improved both the rigor and the clarity of the work. In this revision we have (i) added time-course data on recombinant-protein accumulation by Western blot (two biological replicates) and by whole-plant UV fluorescence imaging; (ii) added a time-resolved qRT-PCR analysis of canonical UPR/ER-stress markers across the buffer and *Agrobacterium* treatment groups; (iii) deposited the raw mass spectrometry data in a public repository; (iv) corrected an erroneous reference and the description of the expression plasmids; and (v) carried out a thorough revision of writing, terminology, Results/Discussion separation, and figure quality. All changes are marked in the tracked-changes manuscript. Point-by-point responses follow.

Response to the Academic Editor

Editor comment: *"It would be important to address Reviewer 1's comments experimentally by providing data at additional time points after infiltration."*

Response: We thank the Editor for this clear guidance, which we have addressed experimentally. We added independent, orthogonal measurements of recombinant-protein accumulation over the infiltration time course:

1. **Western blot (anti-GFP), two independent biological replicates**, sampled at 1, 4 and 7 dpi. In both replicates GFP accumulation rose to a sharp maximum at 4 dpi and declined markedly by 7 dpi (**Response Figure R1B**).
2. **Whole-plant GFP fluorescence under UV illumination** at 1, 4 and 7 dpi, which shows the same pattern at the macroscopic scale (peak green fluorescence at 4 dpi, strongly reduced by 7 dpi; **Response Figure R1A**). As this is a qualitative observation under non-standardized illumination, we present it as visual corroboration in this response letter.
3. A **time-resolved qRT-PCR analysis of ER-stress/UPR markers** (NbBiP, NbPDI, NbbZIP60, NbCRT; see Reviewer 1, comment on lines 266–269) across the buffer, empty-vector and cargo groups at 1, 4 and 7 dpi, provided as **Supplementary Figure S1** and interpreted in the revised Discussion rather than added to the main-text Results.

These results confirm that the decline in recombinant-protein abundance by 7 dpi is a reproducible feature of this *binary T-DNA* expression system rather than an artefact, and they are fully consistent with the MS1-based proteomic time course in Figure 5.

Changes: GFP time-course evidence (anti-GFP Western blot, two biological replicates; whole-plant UV fluorescence) is presented as **Response Figure R1** for the Editor and reviewers and is not incorporated into the manuscript, so no Methods text was added for

it. The UPR-marker qRT-PCR is added as **Supplementary Figure S1** and interpreted in the Discussion, with its procedure described in the Materials and Methods.

Response Figure R1

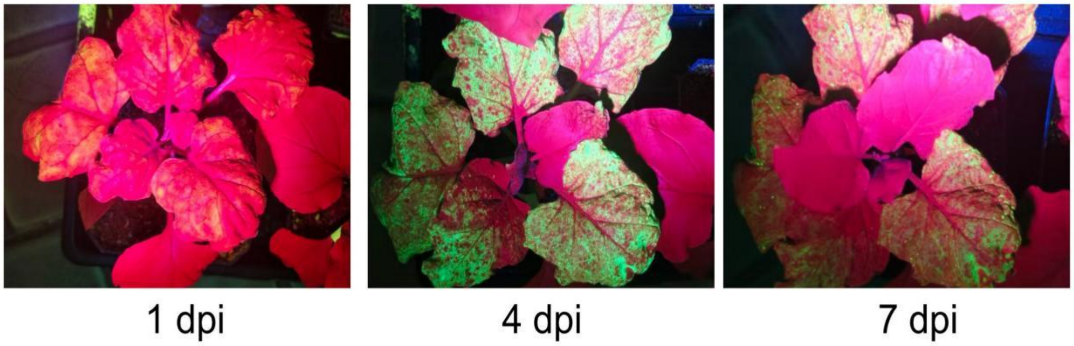

Response Figure R1A — Whole-plant GFP fluorescence under UV illumination at 1, 4 and 7 dpi.

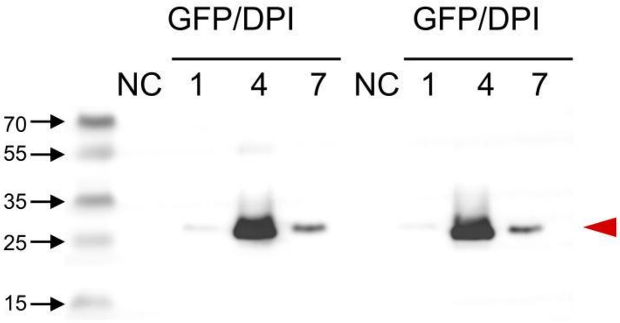

Response Figure R1B — Anti-GFP Western blot of two independent biological replicates at 1, 4 and 7 dpi (NC, non-infiltrated negative control; molecular-mass markers 70/55/35/25/15 kDa; the ~27 kDa GFP band is indicated).

Response Figure R1. GFP accumulation peaks at 4 dpi and declines by 7 dpi in this binary T-DNA system. **(A)** Whole-plant GFP fluorescence under UV illumination at 1, 4 and 7 dpi: green fluorescence is faint at 1 dpi, strongest at 4 dpi, and visibly reduced at 7 dpi. **(B)** Anti-GFP Western blot of two independent biological replicates: the ~27 kDa GFP band is undetectable in the non-infiltrated control (NC), weak at 1 dpi, maximal at 4 dpi, and reduced at 7 dpi in both replicates. Both readouts are concordant with the MS1-based proteomic time course (Figure 5B).

Response to Journal Requirements

JR1 — Style/formatting and file naming. We have reformatted the manuscript and files to the PLOS ONE templates. **Changes:** Title page, headings, and file names conform to the PLOS ONE samples.

JR2 — Code sharing. No custom or bespoke code was used in this study. All analyses were carried out with standard, publicly available software using documented parameters — Spectronaut (v20, Biognosys) for DIA spectral processing, and HMMER (v3.3) with the MEROPS/Pfam databases for protease-domain classification — and the procedures are described in sufficient detail in the Materials and Methods to be reproduced. **Changes:** The Methods specify the software, versions and parameters used; no separate code repository is required.

JR3 — Funding information removed from the manuscript. Changes: The "Funding" sentence has been removed from the manuscript body; funding information is provided only in the online submission Funding Statement.

JR4 — Data availability / non-author contact. To fully comply with the PLOS Data Policy, we have **deposited the raw mass spectrometry proteomics data in a public repository** (ProteomeXchange, accession **PXD080161**, via the iProX partner repository). A non-author contact point is therefore no longer required. **Changes:** The Data Availability statement now reads: *"The mass spectrometry proteomics data have been deposited to the ProteomeXchange Consortium via the iProX partner repository with the dataset identifier PXD080161. The dataset is private during peer review and will be made publicly available on publication; reviewer access can be arranged through the editorial office on request. All other relevant data are within the manuscript and its Supporting Information files."* (This also addresses Reviewer 1's data-availability concern, Q3.)

JR5 — Evaluation of recommended/cited works. We reviewed the cited literature for relevance and accuracy and, in doing so, corrected a bibliographic error (see Reviewer 1, lines 186–190 and our Reference correction below).

Response to Reviewer 1

We thank Reviewer 1 for the detailed and expert reading of our manuscript. The comments identified two genuine weaknesses — an over-interpretation of the cargo-specific stress response, and insufficient attention to the secreted-versus-cytosolic nature of the recombinant proteins — that we have now addressed with new data and a more careful analysis.

Materials and Methods

Comment 1.1 (Line 43): *Xiao et al. (2021) does not present binary plasmids for GFP and FMDV P1_2A in N. benthamiana; this needs to be corrected or the plasmids fully described.*

Response: The reviewer is correct, and we apologize for the imprecise citation. Xiao et al. (2021) was the source of the **FMDV serotype O P1_2A coding sequence only**; the expression constructs themselves were generated in-house. The P1_2A coding sequence, a codon-optimized sequence taken from Xiao et al. (2021) (we did not perform the optimization ourselves), was **chemically synthesized** and cloned into the **pEAQ-HT**

binary vector; the GFP control was cloned into the same backbone; the empty pEAQ-HT vector served as the vector control. We have rewritten the Methods to describe the constructs explicitly and to cite Xiao et al. (2021) appropriately as the sequence source.

Changes: Materials and Methods, "Plant materials and agroinfiltration" revised accordingly.

Results

Comment 1.2 (Lines 133–136): *The text refers to Fig 1D (presence/absence of Agrobacterium) but describes differences linked to the physical act of infiltration.*

Response: We thank the reviewer for catching this inconsistency. The separation highlighted in Fig 1D is between buffer-infiltrated and untreated (WT) tissue — i.e., the effect of the physical infiltration event — not the presence/absence of *Agrobacterium*. We have corrected the text so that the description matches the comparison shown.

Changes: Results reworded to refer to the buffer-vs-WT (mechanical) comparison.

Comment 1.3 (Lines 158–160): *Buffer infiltration induces a stress response that diminishes with time; the authors should consider this in their analysis.*

Response: We agree and have incorporated this explicitly. We now note that the buffer-induced response is strongest early and attenuates over the 1–7 dpi window, and we discuss this temporal decay when interpreting the "background" against which the *Agrobacterium*-specific response is defined.

Changes: Results and Discussion updated to address the time-dependence of the mechanical-stress response.

Comment 1.4 (Lines 186–190): *Prudhomme et al. (2024) studied a secreted antibody (Trastuzumab), which is expected to affect ER protein processing; GFP and FMDV VLPs are not secreted. Results should be presented with this difference in mind.*

Response: We have revised the text accordingly. We now make clear that Prudhomme et al. (2024) examined a **secreted glycoprotein**, whereas the cargoes in our study (GFP and FMDV P1_2A) are **cytosolic and do not transit the secretory pathway**. Because Reviewer 2 also asked that interpretive comparisons be kept out of the Results (comment 2.1), we have moved this point to the Discussion: a dedicated Discussion paragraph now explains the secreted-versus-cytosolic distinction and explicitly cautions against extrapolating secreted-protein ER effects to our cytosolic cargoes, while the Results simply report the observed upregulation of ER protein-processing components.

We also note that, in correcting our citations, we found that the reference we had used for transient UPR induction was bibliographically incorrect: the study by Hamel et al. on influenza haemagglutinin-induced UPR is published in *Plant Biotechnology Journal* (2024) **22:1146–1163, doi:10.1111/pbi.14252**, not in the journal/volume previously cited. This has been corrected.

Changes: Prudhomme ER-processing comparison removed from the Results and the secreted-vs-cytosolic caveat relocated to a dedicated Discussion paragraph (see Reviewer 2, 2.1); Reference list corrected (Hamel et al. 2024).

Comment 1.5 (Lines 199–210): *The comparison with Grosse-Holz et al. (2018) should consider that they studied three secreted proteins; extracellular vs intracellular accumulation may differ.*

Response: We agree. We have added the caveat that Grosse-Holz et al. (2018) characterized the response to **secreted** recombinant proteins and analyzed the **extracellular/secretome** compartment, whereas our total-proteome analysis of **cytosolic** cargoes captures a different protein population. We now frame our comparison around this distinction rather than treating the two settings as equivalent.

Changes: Results and Discussion revised.

Comment 1.6 (Lines 255–258): *"…both recombinant proteins approaching baseline by 7 dpi… regardless of cargo identity." This is reflective of a suboptimal expression system; several publications show continuous GFP accumulation over a week (e.g., Matsuda et al. 2024).*

Response: We thank the reviewer for this important comment, which prompted the additional time-course experiments requested by the Editor. We respectfully suggest that the decline we observe is not evidence of a suboptimal system but a genuine and reproducible property of the **binary T-DNA** platform we used, and that the apparent discrepancy with reports of sustained GFP accumulation reflects a **difference in expression system**:

- Our new **Western blots (two biological replicates)** and **whole-plant UV fluorescence imaging** independently confirm that GFP accumulation peaks at 4 dpi and declines by 7 dpi, in agreement with the MS1-based proteomic time course (**Response Figure R1**).
- Matsuda et al. (2024) used a **viral replicon** (tobamovirus) system, in which continuous replication of the transcript sustains and amplifies reporter accumulation, whereas we used a **binary T-DNA** vector, which is transcribed once and is not amplified. In such non-amplified agroinfiltration systems, an early peak at ~4–5 dpi followed by decline is well documented: (i) in a pEAQ-based reporter-optimization study in *N. benthamiana*, GUS activity peaked at 4 dpi (strains AGL1 and LBA4404) and declined to undetectable levels by 8 dpi (Norkunas et al. 2018); (ii) a pEAQ-expressed pharmaceutical protein (GA733-Fc) reached maximal accumulation at 5 dpi and decreased thereafter (Kim et al. 2021); and (iii) transiently expressed SARS-CoV-2 RBD was highest at 4 dpi and then gradually decreased (Maharjan et al. 2021). The decline we observe by 7 dpi is therefore the expected behaviour of a non-amplified binary T-DNA platform, not a sign of a suboptimal system.
- The two systems are not actually in conflict over the relevant window: Matsuda et al. (2024) themselves report a plateau at ~6–8 dpi, overlapping our 4–7 dpi window; the difference is that ongoing viral replication in their system masks the

host-driven decline, which our non-amplified binary T-DNA system reveals. The vacuum infiltration used here additionally imposes stronger mechanical stress than the syringe infiltration of most cited studies, which would further accentuate the early host response.

We have rewritten this passage to (i) present the new orthogonal data, (ii) attribute the kinetics explicitly to the binary T-DNA system, and (iii) remove any implication of a universal "suppression regardless of cargo."

Changes: Results rewritten; GFP time-course evidence presented as Response Figure R1 (Western blot + UV fluorescence); Discussion contrasts binary T-DNA vs viral-replicon kinetics.

Comment 1.7 (Lines 266–269): *Please explain why P1_2A would exacerbate ER stress and activate UPR-associated degradation.*

Response: This comment identified an over-interpretation on our part, which we have now tested directly and corrected. We performed **time-resolved qRT-PCR of canonical UPR/ER-stress markers** (NbBiP, NbPDI, NbbZIP60, NbCRT) across the buffer, empty-vector (GV3101) and cargo (GFP, P1_2A) groups at 1, 4 and 7 dpi (n = 3). The results show that:

- buffer infiltration does **not** induce these markers (≈1× throughout);
- all *Agrobacterium*-treated groups induce them strongly (≈4–22× over buffer); and
- the magnitude of UPR induction **tracks recombinant-protein accumulation rather than cargo "complexity."** GFP — the high-accumulating cargo — showed the strongest UPR (e.g., NbBiP ≈22× at 4 dpi, coincident with its protein peak), whereas **P1_2A, which accumulates poorly, showed the weakest UPR of the three *Agrobacterium* groups** (NbBiP ≈4× at 4 dpi).

Thus our own data **do not support** a P1_2A-specific exacerbation of ER stress, and we have removed that claim. We also note a mechanistic rationale consistent with the reviewer's broader concern: the reports of VLP/glycoprotein-induced UPR (Hamel et al. 2024, 2025) concern **ER-client secretory glycoproteins** (influenza haemagglutinin; coronavirus spike), whereas GFP and FMDV P1_2A are **cytosolic**, so a cargo-specific UPR is not expected. Accordingly, we now interpret the cargo-associated differences in the protease proteome as **decoupled from the UPR**, and we state that their mechanistic basis remains to be established.

Changes: Removed the ER-stress/UPR mechanistic claim (former lines 266–269); the two references previously cited for that claim (Hatsugai et al. and Thomas) are retained only as a neutral attribution of the vacuolar processing enzymes (VPEs) detected in the protease signature, and are no longer cited in support of a UPR-driven degradation mechanism. We added the UPR-marker qRT-PCR as **Supplementary Figure S1** and describe it in the Discussion (not the main-text Results), reframing UPR as an accumulation-linked, *Agrobacterium*-general response and the cargo-specific protease differences as an observation whose mechanism is not yet resolved.

> **Supplementary Figure S1.** UPR/ER-stress marker transcripts during the infiltration time course. Relative expression of *NbBiP*, *NbPDI*, *NbbZIP60* and *NbCRT* (qRT-PCR; n = 3) in Buffer, GV3101 (empty vector), GFP and P1_2A tissues at 1, 4 and 7 dpi. Buffer remains at ≈1 throughout (mechanical infiltration does not induce the UPR); all *Agrobacterium* groups are strongly induced; induction tracks recombinant-protein accumulation, peaking in GFP at 4 dpi (e.g., *NbBiP* ≈22×) and remaining lowest in the poorly accumulating P1_2A group.

Comment 1.8 (Lines 273–278): *If most tested proteases showed increased transcript when expressing a recombinant protein compared with the GV3101 empty vector, how can these be part of a "general Agrobacterium-responsive program"?*

Response: The reviewer is right that our wording was imprecise. The qRT-PCR data (Fig 5E) show that the tested proteases are induced by *Agrobacterium* relative to buffer **and are further elevated by recombinant-protein expression** (GFP and P1_2A > GV3101 empty vector), without significant differences **between** the two cargoes. We have therefore revised the terminology: these proteases are induced as part of a response to **heterologous protein expression that is independent of cargo identity**, rather than a response to *Agrobacterium* per se. We have ensured this wording is consistent with the proteomic "core" definition and with the empty-vector (GV3101) comparison used throughout.

Changes: Results reworded; terminology harmonized across Results and Discussion.

Discussion

Comment 1.9: *The Discussion should consider that the recombinant proteins here are cytosolic, whereas most other studies evaluated secreted proteins.*

Response: We agree and have added a dedicated paragraph to the Discussion that (i) emphasizes that GFP and FMDV P1_2A are cytosolic, non-secreted proteins; (ii) contrasts this with the secreted/ER-client proteins studied by Prudhomme et al. (2024), Grosse-Holz et al. (2018) and Hamel et al. (2024, 2025); and (iii) explains how this distinction bounds the interpretation of our ER-processing and UPR observations.

Changes: New Discussion paragraph added.

Data availability (Reviewer 1, Q3 = "No"). As noted under JR4, the raw proteomics data have been deposited in the public repository ProteomeXchange (**PXD080161**, via iProX; private during review, public on publication), and the Data Availability statement has been updated.

Response to Reviewer 2

We thank Reviewer 2 for the supportive assessment ("technically sound," sound statistics and data) and for the detailed annotations on the PDF, which we have used to improve

clarity, structure and presentation. We group the annotations by theme below; all individual marks have been addressed in the tracked-changes manuscript.

Comment 2.1 — Results vs Discussion. Several passages in Results read as Discussion. **Response/Changes:** The five highlighted annotations mark four distinct passages (two of the annotations point to the same Prudhomme passage). In every case the interpretive/comparative statement was already (or is now) carried by the Discussion, so we removed it from the Results rather than duplicating it: (i) the "classic wound response" interpretation of the buffer signature (the Discussion already frames this as infiltration stress); (ii) the Grosse-Holz photosynthesis comparison (already in the Discussion); (iii) the Prudhomme ER-processing comparison (consolidated into the new secreted-vs-cytosolic Discussion paragraph, comment 1.4); and (iv) the post-transcriptional-regulation interpretation (verbatim duplicate of an existing Discussion sentence). The Results now report findings only.

Comment 2.2 — Inconsistent terminology ("mock" vs "buffer" vs "WT"). Response: We apologize for the inconsistency. We now use a single, defined set of terms — **"WT"** (untreated), **"buffer"** (buffer-infiltrated, mechanical control), and **"GV3101"** (empty-vector *Agrobacterium*) — and have removed the ambiguous term "mock." **Changes:** Terminology standardized throughout the manuscript and figures.

Comment 2.3 — Figure resolution and a redundant panel. Response: We have re-exported all figures at publication resolution (≥ 300 dpi, TIFF) to meet the PLOS ONE figure specifications, and figure legends are placed in the text immediately after the paragraph in which each figure is first cited, with the figure files supplied separately as required. Regarding Figure 1A (the experimental-workflow schematic), we would respectfully ask to retain it. Although the design is described in the Materials and Methods, this study compares a relatively complex matrix — five treatments (WT, buffer, GV3101, GFP, P1_2A) across three time points in three biological replicates (45 samples), dissecting three distinct stress dimensions — and we have found that the schematic gives readers an immediate visual map of this structure that materially aids interpretation of the subsequent panels and of the buffer-/empty-vector-subtraction logic used throughout. We would, of course, be glad to remove it should the Editor and reviewer still consider it unnecessary. **Changes:** All display items regenerated at full resolution; Figure 1A retained with the justification above; legend placement confirmed against PLOS ONE format.

Comment 2.4 — English language. Response: We have carefully revised the English throughout, including the specific points marked (sub/superscripts in chemical formulae and units, quotation marks, punctuation, word choice, and an inserted reference). **Changes:** Language revised throughout; specific marked items corrected.

Comment 2.5 — Methods (number of PCR reactions per sample). Response: We have added the requested detail. **Changes:** The qRT-PCR Methods now state the number of biological replicates and technical replicates per sample.

Comment 2.6 — Speculation on improving molecular pharming / replacing agroinfiltration. Response: We thank the reviewer for this suggestion, which we found valuable. We have added a short forward-looking paragraph at the end of the Discussion

that (i) outlines how our findings (e.g., targeting specific subtilases/PLCPs; mitigating the early mechanical-stress and accumulation-linked UPR) could improve recombinant-protein yields, and (ii) briefly discusses alternatives to agroinfiltration (e.g., stable lines, viral-replicon or cell-free systems) and their trade-offs. **Changes:** New "speculation / future directions" paragraph added to the Discussion.

Comment 2.7 — Are the proteases biologically validated? Response: We agree this is an important caveat. The protease repertoire is annotated bioinformatically (HMMER/MEROPS) and remains putative; we have stated this explicitly and point to activity-based protein profiling as the appropriate orthogonal validation (already noted as a limitation). **Changes:** Limitation made explicit.

Additional margin annotations. Response: (i) *"Mechanical" vs "osmotic" stress.* The reviewer asked whether the buffer-infiltration stress is mechanical or osmotic. Infiltration in this study was performed by **vacuum infiltration**, in which the reduced pressure imposes a genuine physical/mechanical stress on the leaf tissue; we have therefore retained the term "mechanical" and made this basis explicit in the Methods. (ii) The informal heading "Proteolytic Storm" has been changed to "Proteolytic Response." (iii) "OD_{600}" is now set with a subscript, and the ambiguous term "mock" has been removed (comment 2.2). (iv) *Missing-reference marks.* For the relative-quantification method we have added the standard citation (Livak & Schmittgen, *Methods* 2001, 25, 402–408) after "the 2^(−ΔΔCT) method"; manufacturer city/country has been added throughout the Methods (Bruker Daltonics, Bremen, Germany; Invitrogen, Carlsbad, CA, USA; Vazyme, Nanjing, China; Bio-Rad, Hercules, CA, USA). A primary reference for the phenol-based protein extraction has been added (Li et al., *J. Agric. Food Chem.* 2024, 72, 19526–19536). (v) *Growth light intensity.* The light intensity during plant growth (25,000 lux) has been added to the growth-conditions description. **Changes:** References for the 2^(−ΔΔCT) method (Livak & Schmittgen 2001) and for phenol-based extraction (Li et al. 2024), manufacturer locations, and growth light intensity added; Methods note on vacuum-infiltration mechanical stress added; heading and unit/term edits applied.

Summary of Major Changes

1. **New time-course data on recombinant-protein accumulation** — anti-GFP Western blot (two biological replicates) and whole-plant UV fluorescence imaging at 1/4/7 dpi, presented as **Response Figure R1** (Editor; Reviewer 1, 1.6).
2. **New UPR/ER-stress qRT-PCR** (NbBiP, NbPDI, NbbZIP60, NbCRT; Buffer, GV3101, GFP, P1_2A; 1/4/7 dpi; n = 3), added as **Supplementary Figure S1**, showing UPR scales with protein accumulation and is lowest for P1_2A — removing the previous cargo-specific ER-stress claim (Reviewer 1, 1.7).
3. **Secreted-vs-cytosolic framing** added throughout; comparisons with Prudhomme et al. and Grosse-Holz et al. appropriately bounded; new Discussion paragraph (Reviewer 1, 1.4, 1.5, 1.9).
4. **Corrected plasmid description** (P1_2A sequence from Xiao et al. 2021, synthesized, cloned into pEAQ-HT) and **corrected reference** for Hamel et al. 2024 (Reviewer 1, 1.1, 1.4; JR5).

5. **Terminology, Results/Discussion separation, English, and figure quality** thoroughly revised; figures re-exported to PLOS specifications (Figure 1A retained with justification, comment 2.3) (Reviewer 2).
6. **Raw proteomics data publicly deposited** (ProteomeXchange PXD080161, via iProX); Data Availability and funding statements updated (JR3, JR4; Reviewer 1, Q3).

We believe these revisions strengthen the manuscript and address the points raised, and we thank the Editor and Reviewers again for their time and expertise.

References cited in this response letter (not in the manuscript reference list)

- Matsuda, R.; Urayama, S.; Hosoi, F.; Fujiwara, K. Non-destructive estimation of the amount of green fluorescent protein transiently expressing in *Nicotiana benthamiana* leaves with a viral vector. *Journal of Agricultural Meteorology* **2024**, 80, 29–33.
- Norkunas, K.; Harding, R.; Dale, J.; Dugdale, B. Improving agroinfiltration-based transient gene expression in *Nicotiana benthamiana*. *Plant Methods* **2018**, 14, 71. doi:10.1186/s13007-018-0343-2.
- Kim, K.; Kang, Y.J.; Park, S.R.; Kim, D.-S.; Lee, S.-W.; et al. Effect of leaf position and days post-infiltration on transient expression of colorectal cancer vaccine candidate proteins GA733-Fc and GA733-FcK in *Nicotiana benthamiana*. *PeerJ* **2021**, 9, e10851. doi:10.7717/peerj.10851.
- Maharjan, P.M.; Cheon, J.; Jung, J.; Kim, H.; Lee, J.; et al. Plant-Expressed Receptor Binding Domain of the SARS-CoV-2 Spike Protein Elicits Humoral Immunity in Mice. *Vaccines* **2021**, 9(9), 978. doi:10.3390/vaccines9090978.
