# Supplementary material for: 4D-DIA proteomics reveals distinct proteolytic landscapes induced by mechanical stress, Agrobacterium, and a viral capsid precursor
Source: PLoS One. 2026 Sep 11;21(9):e0356905. doi: 10.1371/journal.pone.0356905 (PMC13567786; doi:10.1371/journal.pone.0356905)
Supplement: Supplementary file 4 [file pone.0356905.s009.docx]

**4D-DIA proteomics reveals distinct proteolytic landscapes induced by mechanical stress, *Agrobacterium*, and complex viral particles**
You Tang^1¶^, Xiaoping Qiu^2¶^, Xinli Ye^2^, Xiaorui Ma^2^, Yilin Mao^2^, Qiwei Yang^2^, Yike Chen^2^, Yiming Sun^2*^, Lijun Zhu^1*^
**1** Chongqing Key Laboratory of Scientific Utilization of Tobacco Resources, Chongqing, China.
**2** School of Life Science, Southwest University, Chongqing, China.
^¶^ These authors (You Tang, Xiaoping Qiu) contributed equally to this work.
* Corresponding author: s8355@swu.edu.cn (Y.S.); zhulj7802@163.com (L.Z.)

# Abstract

*Nicotiana benthamiana* is a widely used platform for plant molecular farming, yet recombinant protein yields are frequently compromised by the host’s innate defense mechanisms, particularly proteolytic degradation. While the general effects of Agroinfiltration are known, the distinct contributions of mechanical injury, bacterial perception, and product-specific stress remain poorly resolved. Here we utilized high-depth 4D-DIA proteomics to dissect the host response across three dimensions: physical stress (buffer infiltration), pathogen-associated stress (*Agrobacterium*), and product-associated stress (GFP vs. FMDV VLPs). We demonstrate that buffer infiltration is not a neutral event but an independent inducer of cell wall remodeling and oxidative stress. By filtering out these background effects, we defined a core Agrobacterium-responsive proteome characterized by a growth-defense trade-off. We also expanded the known protease repertoire of *N. benthamiana* to 1,505 enzymes through improved genomic annotation. We found that the expression of virus-like particles (P1_2A) was associated with a distinct and more pronounced protease profile compared to soluble GFP, characterized by the upregulation of subtilases and cysteine proteases. These findings suggest that host proteolytic responses vary with the recombinant cargo, a factor worth considering when designing engineering strategies for the production of complex biopharmaceuticals in plants or *N. benthamiana*.

# Author summary

Plants are increasingly used as biofactories to produce vaccines and therapeutics. However, a major bottleneck is that plant cells often degrade these valuable recombinant proteins before harvest. While it is widely assumed that this degradation is primarily triggered by the *Agrobacterium* used for gene delivery, we made an unexpected discovery. Using high-resolution 4D-DIA proteomics, we showed that the simple act of buffer infiltration, a routine step in the process, already primes the plant’s defense system, triggering early wound and oxidative stress responses that were previously attributed solely to the bacteria. We also found that complex viral antigens (forming virus-like particles) are subject to a more pronounced and distinct protease response than simple reporter proteins like GFP. This cargo-dependent difference suggests that protection strategies may need to be tailored to the specific product rather than applied uniformly. These findings support the design of product-specific engineering strategies for more practical and affordable plant-based medicine production.

# Introduction

Plant molecular farming (PMF) is defined as the use of plant-based platforms to produce valuable proteins or metabolites that are used in the biopharmaceutical, nutraceutical or cosmetic industries, and more. *Nicotiana benthamiana* is the preferred host due to its susceptibility to transfection, rapid growth, and high biomass generation [1]. Despite these advantages, the accumulation of exogenously expressed proteins is often limited by the plant’s physiological transition from a growth state to a defense state upon agroinfiltration [2]. This defense response creates a proteolytic environment in the apoplast and intracellular compartments, leading to product degradation and reduced yields [3][4]. Although strategies such as co-expression of protease inhibitors have mitigated some losses [5][6], current approaches often rely on a generalized understanding of stress rather than addressing specific degradation pathways. The stress imposed during transient expression is cumulative and multi-layered. The infiltration process itself applies significant mechanical pressure to leaf tissue, potentially triggering wound responses that are distinct from the immune responses elicited by *Agrobacterium tumefaciens’* pathogen-associated molecular patterns (PAMPs) [7].

The physicochemical properties of the recombinant product influence the magnitude of cellular stress. Complex proteins that transit the secretory pathway, such as enveloped virus-like particles (VLPs), require extensive folding and assembly machinery within the endoplasmic reticulum (ER). Recent transcriptomic studies suggest that VLP expression triggers the unfolded protein response (UPR) and immune signaling more intensely than simple soluble proteins like Green Fluorescent Protein (GFP) [8][9]. Whether cytosolic cargoes that do not enter the secretory pathway impose a comparable burden remains unclear, and proteomic evidence distinguishing these layers of stress is limited. To address these complexities, we employed 4D-DIA (Data-Independent Acquisition) mass spectrometry, which incorporates ion mobility separation to achieve superior sensitivity and proteome coverage compared to traditional methods [10].

We designed an experiment to systematically decouple the sources of host stress by comparing non-infiltrated wild-type plants (WT), buffer-infiltrated controls (mechanical stress), empty vector *Agrobacterium* (bacterial stress), and *Agrobacterium* expressing either GFP or the Foot-and-Mouth Disease Virus antigen P1_2A (product-specific stress) [11]. This study provides a high-resolution map of the *N. benthamiana* proteome dynamics, revealing that the host proteolytic landscape is actively reshaped by the specific nature of the recombinant cargo. We aimed to: (1) determine whether buffer infiltration alone induces a distinct proteomic signature separate from the *Agrobacterium*-mediated immune response; (2) define the core *Agrobacterium*-responsive proteome independent of cargo effects; and (3) test the hypothesis that host proteolytic responses are cargo-dependent, with complex viral antigens eliciting a more pronounced protease response than simple soluble proteins.

# Materials and methods

## Plant material and Agroinfiltration

Wild-type *N. benthamiana* plants were grown in chambers at 25 °C under a 16 h light/8 h dark photoperiod at a light intensity of 25,000 lux. The coding sequences for GFP and the FMDV P1_2A capsid precursor were each cloned into the binary vector pEAQ-HT, which co-expresses the P19 silencing suppressor; the P1_2A coding sequence, a codon-optimized sequence based on Xiao et al. [11], was chemically synthesized. These constructs, together with empty pEAQ-HT as a vector control, were introduced into *Agrobacterium tumefaciens* strain GV3101 and cultured in LB medium. Cells were resuspended in infiltration buffer (10 mM MES, 10 mM MgCl₂, 100 µM acetosyringone) and adjusted to an OD_600_ of 0.6. Infiltration was performed using a vacuum-infiltration system (PTS-50, WONBIO, Shanghai, China) on leaves of 5-week-old plants according to Yao et al. [12].

## Proteomic analysis (4D-DIA)

Leaf samples were harvested at 1, 4, and 7 dpi (days post agro-infiltration), flash-frozen in liquid nitrogen, and ground to a fine powder. Total proteins were extracted using a phenol-based method [13]. Protein concentration was determined using a BCA assay, and protein integrity was verified by sodium dodecyl sulfate-polyacrylamide gel electrophoresis (SDS-PAGE). Samples were shipped on dry ice to Majorbio Biomedical Technology Co., Ltd. (Shanghai, China) for LC-MS/MS analysis. Proteins were digested with trypsin (enzyme:substrate ratio of 1:50 w/w) at 37 °C for 16 h. The resulting peptides were desalted and separated on a nanoElute UHPLC system (Bruker, Germany) equipped with a C18 analytical column (75 μm × 25 cm, 1.6 μm particle size) at a flow rate of 300 nL/min over a 60-min linear gradient. Mobile phase A consisted of 98% water, 2% acetonitrile, and 0.1% formic acid; mobile phase B consisted of 80% acetonitrile, 20% water, and 0.1% formic acid. Data acquisition was performed on a timsTOF Pro mass spectrometer (Bruker Daltonics, Germany) operating in 4D-DIA mode, which leverages trapped ion mobility spectrometry (TIMS) to separate ions based on their collisional cross-section, providing an additional dimension of separation beyond retention time, m/z, and intensity[10]. The MS1 scan range was set to m/z 100-1700, and the TIMS ramp time was 100 ms with an ion mobility range (1/K0) of 0.6-1.6 V·s/cm².

## Database search and quantification

Raw data were processed with Spectronaut (version 20) and searched against the *N. benthamiana* proteome database (HZ version 1) [14], to which the GFP and FMDV P1_2A transgene sequences were appended. Identifications were filtered at a false discovery rate (FDR) ≤ 1% at both the peptide and protein-group levels, with a minimum peptide confidence of 99% and an XIC extraction window of ≤ 75 ppm. In total, 61,379 unique peptide sequences were identified, yielding 9,495 protein groups quantified across the 45 samples. The mass spectrometry proteomics data have been deposited to the ProteomeXchange Consortium via the iProX partner repository with the dataset identifier PXD080161.

## Bioinformatics analysis

A reference database containing specific Pfam domain IDs associated with proteases was constructed, referencing established classifications [15]. The protein sequences of the latest *N. benthamiana* genome HZ version 1 [14] were scanned for functional domains using hmmer (version 3.3) with default E-value thresholds (sequence E-value < 10, domain E-value < 10). Proteins were categorized by matching their identified Pfam domains against the reference dataset (Table S3). Prior to differential expression analysis, an abundance-based pre-filtering step was applied. The first quartile (Q1) of protein abundances across all control samples (WT and buffer groups at all time points) was calculated as a baseline threshold. Only proteins with a maximum abundance exceeding this Q1 value in at least one control sample were retained for subsequent identification of differentially expressed proteins (DEPs). DEPs were identified using pairwise Student’s t-tests between treatment groups and controls. Proteins with an absolute fold change > 1.5 and a Benjamini-Hochberg adjusted P-value (FDR) < 0.05 were defined as significantly differentially expressed. The experimental design comprised five treatment groups (WT, Buffer, GV3101, GFP, and P1_2A) sampled at three time points (1, 4, and 7 dpi), with three independent biological replicates per condition, yielding a total of 45 samples. To determine the biological functions of the DEPs in each comparison, functional enrichment analyses were performed encompassing Gene Ontology (GO) terms, KOG classifications, KEGG pathways, and protein domains. A hypergeometric test was employed to calculate the statistical significance (P-value), identifying specific functional categories that were significantly over-represented among the DEPs relative to the entire identified proteome as the background.

## Real-time quantitative PCR analysis

Total RNA was isolated from *N. benthamiana* leaves using TRIzol Reagent (Invitrogen, USA) and subsequently treated with DNase I to eliminate genomic DNA contamination. First-strand cDNA was synthesized employing the HiScript^®^ II Q RT SuperMix (+gDNA wiper) kit (Vazyme, China). Real-time quantitative PCR (RT-qPCR) was performed with three technical replicates per sample on a CFX Connect Real-Time PCR Detection System (Bio-Rad, USA) utilizing SYBR Green chemistry. The thermocycling parameters consisted of 40 cycles at 95 °C for 10 s and 60 °C for 30 s, with fluorescence signals acquired at the end of each cycle. Relative gene expression levels were determined via the 2^−ΔΔCt^ method [16], utilizing *β-actin* as the reference gene. In addition to the ten protease genes, four canonical UPR/ER-stress markers (NbBiP, NbPDI, NbbZIP60 and NbCRT) were assessed by the same procedure across the buffer, empty-vector (GV3101), GFP and P1_2A groups at 1, 4 and 7 dpi (S1 Fig). Primer sequences are listed in Table S4. All analyses were conducted with three biological replicates.

# Results

## Deep proteome coverage via 4D-DIA captures subtle host dynamics

We utilized the 4D-DIA platform to analyze the temporal dynamics of the *N. benthamiana* proteome at 1, 4, and 7 dpi. The experimental design included five treatment groups to isolate specific stress factors (Fig 1A). A total of 61,379 unique peptide sequences were identified from the raw data and mapped to 9,495 protein groups across three independent biological replicates and subsequently quantified using the MS1 intensity-based label-free quantification (LFQ) method (Table S1). Pearson correlation analysis confirmed high reproducibility across all 45 proteomes, with average correlation coefficients exceeding 0.95 within each treatment group (Fig 1B). UMAP dimensionality reduction of the global proteome revealed a clear topological separation between agroinfiltrated and non-infiltrated samples: WT and buffer controls formed a distinct cluster from the GV3101, GFP, and P1_2A groups, and this divergence intensified progressively from 1 to 7 dpi (Fig 1C). This global trajectory demonstrates that *Agrobacterium*-mediated transformation fundamentally reshapes the host proteome in a time-dependent manner. In the per-time-point PCA (Fig 1D), the buffer-infiltrated group separated clearly from the untreated WT group as early as 1 dpi, and this separation persisted at 4 dpi. As buffer and WT plants differ only in the mechanical act of infiltration, neither receives Agrobacterium, this shift specifically reflects the wounding/mechanical component of the response, as distinct from the larger *Agrobacterium*-driven reprogramming.

**Fig 1. Experimental design and global proteomic landscape of *N. benthamiana* responses to agroinfiltration.** (A) Experimental workflow. *N. benthamiana* leaves were subjected to five treatments: untreated wild type (WT), buffer control, empty *Agrobacterium* (GV3101), and *Agrobacterium* expressing GFP or FMDV antigen P1_2A. Samples were harvested at 1, 4, and 7 days post-infiltration (dpi) and analyzed via 4D-DIA LC-MS/MS (n=3 biological replicates). (B) Pearson correlation heatmap of all 45 proteomes. (C) UMAP visualization of the global proteomic trajectory, showing temporal shifts and distinct clustering between non-infiltrated controls (WT/buffer) and *Agrobacterium*-infiltrated groups. (D) PCA plots at individual time points (1, 4, and 7 dpi).

## Buffer infiltration triggers early defense and wound responses

To understand the "background noise" in molecular farming experiments, we analyzed the DEPs between the Buffer and WT groups. The volcano plots revealed that the buffer treatment is biologically active, inducing significant protein changes as early as 1 dpi (Fig 2A, Table S2). Functional enrichment analysis (GO and KEGG) indicated a significant upregulation of proteins involved in phenylpropanoid biosynthesis, MAPK signaling, and oxidoreductase activity (Fig 2B). Specifically, terms such as "cell wall biogenesis", "apoplast", and "hydrogen peroxide catabolic process" were enriched (Fig 2C).

**Fig 2. Time-course proteomic profiling and functional enrichment in response to buffer infiltration.** (A) Volcano plots of differentially expressed proteins (DEPs) between buffer-infiltrated and untreated WT leaves at 1, 4, and 7 days post-infiltration (dpi). Red and blue dots indicate significantly up- and down-regulated proteins, respectively (fold change > 1.5, P < 0.05). (B) KEGG pathway enrichment analysis of the up- and down-regulated DEPs at 1, 4, and 7 dpi. (C) Gene Ontology (GO) enrichment analysis of biological processes and molecular functions for the up- and down-regulated DEPs at 1, 4, and 7 dpi.

## The core *Agrobacterium*-responsive proteome

Having established that buffer infiltration alone constitutes a significant abiotic stressor, we next sought to identify the proteomic changes attributable specifically to *Agrobacterium*. To isolate the host response specifically attributable to *Agrobacterium* infection, we identified the intersection of DEPs found in all bacteria-treated groups (GV3101, GFP, and P1_2A) relative to the Buffer control (Fig 3A, Table S2). This approach filters out mechanical stress, leaving a "core *Agrobacterium*-responsive proteome." This core response was characterized by extensive metabolic reprogramming consistent with the growth-defense trade-off hypothesis [17]. We observed a universal downregulation of proteins associated with photosynthesis (e.g., photosynthesis, light harvesting, chlorophyll A-B binding proteins, and Rubisco small subunits) and carbon metabolism across all time points (Fig 3B, 3C). Conversely, proteins involved in protein processing in the endoplasmic reticulum (ER), ribosome biogenesis, and vesicle trafficking were significantly upregulated (Fig 3B). KEGG pathway analysis further confirmed this metabolic shift, revealing the downregulation of photosynthesis-antenna proteins and carbon fixation pathways, while plant-pathogen interaction, MAPK signaling, and protein processing in the ER were enriched among the upregulated core proteins (Fig 3C). The enrichment of plant-pathogen interaction pathways is consistent with PAMP perception via pattern recognition receptors such as EFR, which detects the bacterial elongation factor EF-Tu [18]. At the metabolic level, Prudhomme et al. [19] demonstrated that the jasmonic acid (JA) precursor 12-OPDA and six fatty acid β-oxidation enzymes accumulated in agroinfiltrated tissue, connecting PAMP-triggered immunity to hormonal defense signaling. The extracellular proteome analysis by Grosse-Holz et al. [20] showed that hydrolytic defense proteins, including chitinases (GH18), PR2 glucanases (GH17), and xylanase inhibitors, accumulated in the apoplast by 2 dpi, while papain-like cysteine proteases (PLCPs, family C01) did not increase proportionally in the extracellular fraction. In contrast, our total cellular proteome data captured the intracellular accumulation of these PLCPs, suggesting that many PLCPs induced during agroinfiltration are retained in vacuolar or ER-associated compartments rather than being secreted. The number of core DEPs increased from 1 to 7 dpi, indicating a progressive amplification of the host defense response over time. This temporal escalation is consistent with the pattern reported by Grosse-Holz et al. [20], in which the number of differentially abundant extracellular proteins expanded from 2 to 10 dpi.

**Fig 3. Identification and functional enrichment of the core *Agrobacterium*-responsive proteome.** (A) Venn diagrams displaying the overlap of DEPs among GV3101, GFP, and P1_2A treatments relative to buffer control at 1, 4, and 7 dpi. (B) Gene Ontology (GO) enrichment analysis of the up- and down-regulated core DEPs. (C) KEGG pathway enrichment analysis.

## Expanded protease landscape and the core proteolytic response

Proteases are the primary bottleneck in molecular farming. Given that proteolytic degradation is a primary barrier to recombinant protein accumulation, we next focused our analysis on the protease component of these responses. Leveraging the latest *N. benthamiana* genome assembly [15] and our deep proteomic data, we annotated 1,505 putative proteases, significantly expanding upon the 1,243 proteases previously reported [20]. These were classified into catalytic families based on MEROPS nomenclature [21], with serine (Ser), cysteine (Cys), and aspartic (Asp) proteases dominating the landscape (Fig 4A, Table S3). Hierarchical clustering showed that a large subset of these proteases is upregulated upon agroinfiltration regardless of the cargo (Fig 4B). We identified specific enzymes, such as the aspartic protease NbALP1, the cysteine protease NbCTB2, and the subtilase NbSBT6, as part of the core proteolytic response (Fig 4C). These enzymes showed consistent upregulation in GV3101, GFP, and P1_2A groups compared to buffer controls. NbALP1 and NbCTB2 are known to be involved in the degradation of recombinant proteins in the apoplast [22][23].

**Fig 4. The expanded protease landscape of *N. benthamiana* and the core proteolytic response.** (A) Classification of the 1,505 proteases identified in this study into MEROPS catalytic classes (Serine, Cysteine, Aspartic, Metallo, Threonine). (B) Hierarchical clustering of protease expression profiles across all treatments. Red indicates upregulation; blue indicates downregulation. (C) Expression profiles of representative "Core" proteases including NbALP1 (Aspartic), NbCTB2 (Cysteine), NbSBT6 (Serine), and NbVPE1b (Cysteine).

## P1_2A triggers a product-specific proteolytic response

While the above analyses identified a common proteolytic response to *Agrobacterium*, a key question for molecular farming is whether the nature of the recombinant cargo further shapes the host response. A direct comparison between the GFP and P1_2A expression groups revealed marked differences in the host response. P1_2A, an animal viral capsid precursor that self-assembles into VLPs (Ref`??), induced a significantly higher number of DEPs compared to the soluble GFP reporter, particularly at 4 dpi (Fig 5B, Table S2). Functional enrichment analysis of the P1_2A-specific response highlighted pathways related to cytoskeleton organization ("microtubule-based process"), nuclear pore complex, and chromatin remodeling (Fig 5A). This likely reflects the complex cellular requirements for VLP assembly and trafficking [8]. Most critically, P1_2A expression triggered the specific and intense upregulation of a distinct subset of proteases (Fig 5D). Time-course analysis of recombinant protein accumulation revealed markedly different dynamics between the two cargoes: GFP protein intensity peaked sharply at 4 dpi before declining by 7 dpi, whereas P1_2A protein levels remained consistently low throughout the time course (Fig 5B). A similar trend was observed for relative expression levels, with both recombinant proteins approaching baseline by 7 dpi (Fig 5C). While GFP achieved substantially higher peak accumulation, the P1_2A group exhibited a more sustained and intense stress-related proteome. The heatmap of cargo-specific differentially expressed proteases revealed that 10 proteases spanning three catalytic classes [aspartic (Asp), cysteine (Cys), and serine (Ser)] were significantly differentially regulated between GFP and P1_2A at 4 dpi (Fig 5D). Specific proteases, including NbSBT9 (serine), NbRD21B (cysteine), and NbVPE1b (Vacuolar Processing Enzyme, cysteine), were hyper-induced in the P1_2A group, along with previously uncharacterized proteases Nbe16g06900.1 (Asp), Nbe19g17120.1 (Cys), and Nbe14g08810.1 (Cys). This defines a cargo-specific protease signature at 4 dpi that includes vacuolar processing enzymes (VPEs) [24][25]. As shown below, however, this signature was not accompanied by cargo-specific differences at the transcript level, suggesting that it may reflect differences in the level or kinetics of recombinant-protein accumulation rather than a distinct P1_2A-specific stress program.

## RT-qPCR validation of cargo-specific protease induction

To validate the proteomic findings at the transcript level, we performed quantitative real-time PCR (RT-qPCR) analysis on ten selected protease genes that were differentially expressed in the P1_2A group (Fig 5E). The majority of the tested proteases, including *NbSBT9* (2.29-fold), *NbRD21B* (2.53-fold, *P*= 0.049), *NbALP1* (2.84-fold, *P*= 0.014), and *Nbe16g06900.1* (2.18-fold), showed elevated transcript levels in both the GFP and P1_2A groups relative to the GV3101 empty vector control, indicating that their induction is driven by recombinant-protein expression itself rather than by *Agrobacterium* alone, and that it does not differ between the two cargoes. *NbRD21B* and *Nbe16g06900.1* exhibited statistically significant upregulation in the P1_2A group compared to GV3101 (*P*= 0.006 and *P*= 0.003, respectively). However, the transcript-level differences between P1_2A and GFP groups did not reach statistical significance for any of the tested genes (*P*> 0.05), despite clear differences at the protein level (Fig 5D). Two genes, *Nbe19g17120.1* (1.60-fold, *P*= 0.081) and *Nbe11g00200.1* (1.52-fold, *P*= 0.092), showed a trend toward higher expression in the P1_2A group but remained below the significance threshold.

**Fig 5. Cargo-specific proteomic responses and RT-qPCR validation.** (A) Gene Ontology (GO) functional enrichment analysis of differentially expressed proteins between P1_2A and GFP groups at 4 dpi. Node color indicates -log10(P-value) and node size indicates the protein ratio. (B) Time-course comparison of relative protein intensity for the recombinant proteins GFP and P1_2A at 1, 4, and 7 dpi (mean ± SD, n=3). (C) Relative expression levels of GFP and P1_2A over the infiltration time course, normalized to 1 dpi values. (D) Heatmap of cargo-specific differentially expressed proteases between GFP and P1_2A groups at 4 dpi. Proteases are classified by catalytic type: Asp (aspartic), Cys (cysteine), and Ser (serine). Red indicates upregulation; blue indicates downregulation. (E) qRT-PCR validation of ten selected protease genes. Bar plots show relative expression levels in GV3101, GFP, and P1_2A groups at 4 dpi (mean ± SD, n=3). Asterisks indicate statistical significance (*P < 0.05, Student’s t-test).

# Discussion

Our study provides a high-resolution, temporally resolved proteomic atlas of the multi-layered host response during agroinfiltration of *N. benthamiana*, revealing that the widely assumed "neutral" controls (TO NAME THEM) in molecular farming are biologically active. The buffer infiltration alone induced significant changes in the proteome, including the upregulation of cell wall remodeling enzymes, MAPK signaling components, and oxidoreductases as early as 1 dpi (Fig 2). This finding is consistent with the "infiltration stress" concept described by Hamel [2] and the metabolic perturbations reported by Drapal et al. [7] in different *N. benthamiana* accessions. The activation of phenylpropanoid biosynthesis pathways in our buffer-infiltrated samples mirrors the wound-induced lignification response documented in other systems. Prudhomme et al. [19] similarly noted that bacterial growth alone reshapes the *N. benthamiana* metabolome, but our data extend this by showing that even the mechanical component of infiltration, without any bacterial presence, is sufficient to prime the defense transcriptome. This has practical implications: experiments using buffer-infiltrated controls as a "baseline" may systematically underestimate the total stress burden imposed on the plant, as these controls already represent a perturbed state.

By subtracting the buffer infiltration background, we defined a core *Agrobacterium*-responsive proteome that was shared across all bacteria-treated groups regardless of the expressed cargo. This core response was dominated by a growth-defense trade-off, with photosynthetic proteins consistently downregulated and defense-related proteins upregulated (Fig 3). This pattern aligns closely with the transcriptomic findings of Grosse-Holz et al. [20], who reported a similar suppression of photosynthesis-related transcripts during agroinfiltration. Our proteomic data confirm that this trade-off is also manifest at the protein level and persists across the 1–7 dpi time window. The metabolic cost of mounting this defense was recently shown to be partially reversible through light and cytokinin co-treatments [17], which underlines the need to understand these basal responses for yield optimization. The upregulation of ER-associated protein processing and vesicle trafficking components in the core response suggests that PAMP recognition by receptors such as EFR [18] triggers a sustained secretory pathway remodeling, which may compete with recombinant protein folding and secretion.

IS THERE A BUFFER AND AGROBACTERIUM CONTROLS INDUCED PROTEIN DIFFERNING IN THE COMPARISON OF GFP VS. P1_2A (CARGO-MEDIATED) PROTEOMES?

Perhaps the most consequential finding for the molecular farming is the demonstration that host proteolytic responses are not uniform but cargo-dependent. The P1_2A viral capsid precursor induced a quantitatively and qualitatively distinct protease profile compared to soluble GFP (Fig 5D). The specific upregulation of subtilases (e.g., NbSBT9), papain-like cysteine proteases (e.g., NbRD21B), and vacuolar processing enzymes (e.g., NbVPE1b) in the P1_2A group points to the activation of both apoplastic and vacuolar degradation pathways. We initially considered whether this cargo-specific difference might reflect differential ER stress, by analogy to reports that secretory glycoproteins such as influenza haemagglutinin and coronavirus-like particles trigger an intense UPR in *N. benthamiana* [8][9]. However, those cargoes are ER-resident secretory glycoproteins, whereas GFP and the FMDV P1_2A capsid precursor are cytosolic proteins that do not transit the secretory pathway. Consistent with this, the UPR-marker RT-qPCR (NbBiP, NbPDI, NbbZIP60, NbCRT) showed that the UPR was broadly induced by agroinfiltration but scaled with recombinant-protein accumulation rather than with cargo identity, being lowest in the poorly accumulating P1_2A group, indicating that the cargo-specific protease differences are unlikely to be driven by a P1_2A-specific UPR (S1 Fig). Grosse-Holz et al. [20] identified approximately 1,243 proteases from their transcriptome and secretome analyses; our 4D-DIA approach expanded this repertoire to 1,505 enzymes, likely reflecting the improved sensitivity afforded by ion mobility separation and the use of an updated genome assembly. Our RT-qPCR data revealed that the cargo-specific differences in protease abundance are not fully reflected at the transcript level, as P1_2A vs. GFP comparisons did not reach statistical significance for any of the ten tested protease genes (Fig 5E). This discordance suggests that post-transcriptional regulation, such as differential mRNA stability or protein turnover, plays a significant role in shaping the cargo-specific proteolytic landscape. These findings argue against the "one-size-fits-all" approach to protease inhibitor co-expression and instead support targeted strategies: for complex antigens like P1_2A, silencing or inhibiting specific SBTs and PLCPs [6][26] may be more effective than broad-spectrum approaches. The 4D-DIA approach enabled the identification of 9,495 protein groups across all samples, achieving a deeper proteome coverage than previous DDA-based studies of agroinfiltrated *N. benthamiana*. The incorporation of ion mobility as a fourth separation dimension enhanced peptide identification confidence and quantification accuracy, particularly for low-abundance regulatory proteins such as proteases and signaling kinases. The annotation of 1,505 putative proteases, representing a 21% increase over the 1̃,243 reported by Grosse-Holz et al. [20], can be attributed to three factors: (i) the use of the updated HZ version 1 genome assembly [14], which provides more complete gene models; (ii) the superior sensitivity of 4D-DIA in detecting low-abundance proteins; and (iii) systematic hmmer-based domain scanning against the MEROPS/Pfam reference database with default E-value thresholds. However, some of these annotations remain putative and would benefit from experimental validation of proteolytic activity, for instance through activity-based protein profiling as described by Jutras et al. [22]. This study has several limitations. First, our analysis was conducted using a single *N. benthamiana* accession under controlled growth conditions; the generalizability of these findings to other accessions or field-grown plants remains to be established [7]. Second, we sampled at three time points (1, 4, and 7 dpi), which captures the major temporal dynamics but may miss early signaling events occurring within hours of infiltration. Third, we used a single *Agrobacterium* density (OD₆₀₀ = 0.6); dose-dependent effects on the host proteome were not explored. Fourth, while our RT-qPCR validation targeted ten protease genes, confirmation of the broader proteomic findings would benefit from orthogonal approaches such as Western blotting or targeted proteomics (PRM/SRM). Finally, the mechanistic basis of the cargo-specific protease induction, whether driven by ER stress sensing, T-DNA integration efficiency, or direct protein–protein interactions, is not yet clear. Future studies combining genetic perturbation (e.g., CRISPR-mediated knockout of key SBTs and PLCPs) with time-resolved proteomics will be essential to translate these observations into actionable strategies for improving recombinant protein yields in plant molecular farming.

The observation that recombinant-protein levels peaked at 4 dpi and declined toward baseline by 7 dpi should not be interpreted as evidence of a suboptimal expression system. In the binary T-DNA system used here, each transgene is transcribed only once from the integrated T-DNA and is not subsequently amplified, so product accumulation is expected to rise to an early maximum and then fall as the host defense response develops and the infiltrated tissue ages. This contrasts with viral-replicon (deconstructed-virus) vectors, in which continuous replication of the transgene transcript sustains and amplifies accumulation over a longer window; reports of sustained recombinant-protein accumulation over a week therefore reflect the replicative nature of those systems rather than a general property of agroinfiltration. The 4 dpi peak we observe is thus consistent with the intrinsic kinetics of non-amplified transient expression, and the cargo-independent decline reflects this shared kinetic ceiling rather than a distinct, cargo-specific suppression mechanism.

Our results also point to practical routes for improving plant-based recombinant-protein production. Because the cargo-specific losses we observe track particular host proteases rather than a generalized stress program, suppressing the specific subtilases and papain-like cysteine proteases induced during expression—rather than co-expressing broad-spectrum protease inhibitors—may be a more effective way to stabilize complex antigens such as P1_2A. Reducing the early, infiltration-associated mechanical stress and the accumulation-linked UPR, for instance through gentler delivery, optimized infiltration media, or co-expression of stress-buffering factors, may extend the productive window beyond the 4 dpi peak seen here. The question of how agroinfiltration itself might be replaced is also pertinent: stable transgenic lines avoid the acute wound-and-immune response but need much longer development times; viral-replicon vectors sustain higher per-cell expression through transcript amplification but raise biosafety and genetic-stability concerns; and cell-free or suspension-culture platforms trade scale and post-translational capacity for greater control. A host-proteome map of the kind presented here offers a rational starting point for choosing and engineering among these options.

A broader consideration when interpreting our results is the subcellular destination of the recombinant cargoes. GFP and the FMDV P1_2A capsid precursor are cytosolic proteins that are neither translocated into the endoplasmic reticulum nor routed through the secretory pathway, whereas much of the molecular-farming literature against which we benchmark our data concerns secreted or ER-resident products, for example the secreted antibody trastuzumab studied by Prudhomme et al. [19], the three secreted recombinant proteins profiled by Grosse-Holz et al. [20], and the secretory glycoprotein VLPs such as influenza hemagglutinin and coronavirus-like particles that elicit a strong UPR [8][9]. Secreted and ER-client proteins place folding, glycosylation and quality-control demands directly on the ER, so the ER-processing and UPR responses described in those systems may not operate identically for the cytosolic cargoes used here. This difference bounds how far our observations can be compared with that body of work, and it is consistent with our finding that the cargo-specific protease differences are decoupled from a classical, secretion-linked UPR. It also implies that engineering strategies aimed at relieving ER stress during secreted-protein production may transfer only partially to cytosolic antigens such as P1_2A.

# Conclusion

In conclusion, this study provides three main insights. First, buffer infiltration is not a neutral control but actively primes wound and oxidative stress responses, confounding the interpretation of bacterial and product-specific effects. Second, the core Agrobacterium-responsive proteome is defined by a sustained growth-defense trade-off, explaining the metabolic cost of transient expression. Third, host proteolytic responses vary with the recombinant cargo: the complex VLP antigen P1_2A was associated with a distinct and more pronounced protease signature—enriching subtilases and cysteine proteases—that was not mirrored by proportional cargo-specific differences at the transcript level. These findings argue against a uniform, broad-spectrum protease-inhibitor strategy and instead support approaches tailored to the target product.

# Supporting information

#### **S1 Fig. UPR/ER-stress marker expression during the infiltration time course.** Relative expression of NbBiP, NbPDI, NbbZIP60 and NbCRT (qRT-PCR; n = 3) in buffer, GV3101 (empty vector), GFP and P1_2A tissues at 1, 4 and 7 dpi. Buffer remains near baseline throughout, whereas all Agrobacterium groups are induced; induction tracks recombinant-protein accumulation, peaking in GFP at 4 dpi and remaining lowest in the poorly accumulating P1_2A group.

#### **S1 Table. Summary of proteomic data acquisition and identification.**

#### **S2 Table. Summary of differentially expressed proteins (DEPs) between Buffer and WT groups at 1, 4, and 7 days post-infiltration (dpi).**

#### **S3 Table. Annotation of putative proteases identified in Nicotiana benthamiana with MEROPS-based classification and Pfam domain information.**

**S4 Table. Primers used for RT-qPCR.**

**References**

1.	J. Bally, H. Jung, C. Mortimer, F. Naim, J. G. Philips, R. Hellens, A. Bombarely, M. M. Goodin, P. M. Waterhouse. The rise and rise of *Nicotiana benthamiana*: A plant for all reasons. Annu Rev Phytopathol. 2018;56:405–26. doi:[10.1146/annurev-phyto-080417-050141](https://doi.org/10.1146/annurev-phyto-080417-050141)

2.	L. Hamel. *Nicotiana benthamiana*’s responses to agroinfiltration, a treasure grove of new avenues to improve protein yields in plant molecular farming. Plant Biotechnol J. 2026;24:5–17. doi:[10.1111/pbi.70460](https://doi.org/10.1111/pbi.70460)

3.	P. V. Jutras, I. Dodds, R. A. van der Hoorn. Proteases of *Nicotiana benthamiana*: An emerging battle for molecular farming. Curr Opin Biotechnol. 2020;61:60–5. doi:[10.1016/j.copbio.2019.10.006](https://doi.org/10.1016/j.copbio.2019.10.006)

4.	M. K. Mandal, H. Ahvari, S. Schillberg, A. Schiermeyer. Tackling unwanted proteolysis in plant production hosts used for molecular farming. Front Plant Sci. 2016;7. doi:[10.3389/fpls.2016.00267](https://doi.org/10.3389/fpls.2016.00267)

5.	C. Goulet, M. Khalf, F. Sainsbury, M. A. D’Aoust, D. Michaud. A protease activity-depleted environment for heterologous proteins migrating towards the leaf cell apoplast. Plant Biotechnol J. 2012;10:83–94. doi:[10.1111/j.1467-7652.2011.00643.x](https://doi.org/10.1111/j.1467-7652.2011.00643.x)

6.	F. Grosse-Holz, L. Madeira, M. A. Zahid, M. Songer, J. Kourelis, M. Fesenko, S. Ninck, F. Kaschani, M. Kaiser, R. A. L. van der Hoorn. Three unrelated protease inhibitors enhance accumulation of pharmaceutical recombinant proteins in *Nicotiana benthamiana*. Plant Biotechnol J. 2018;16:1797–810. doi:[10.1111/pbi.12916](https://doi.org/10.1111/pbi.12916)

7.	M. Drapal, E. M. A. Enfissi, P. D. Fraser. Metabolic effects of agro-infiltration on n. Benthamiana accessions. Transgenic Research. 2021;30:303–15. doi:[10.1007/s11248-021-00256-9](https://doi.org/10.1007/s11248-021-00256-9)

8.	L. P. Hamel, M. A. Comeau, R. Tardif, F. Poirier-Gravel, M. Paré, P. O. Lavoie, M. C. Goulet, D. Michaud, M. A. D’Aoust. Heterologous expression of influenza haemagglutinin leads to early and transient activation of the unfolded protein response in *Nicotiana benthamiana*. Plant Biotechnol J. 2024;22:1146–63. doi:[10.1111/pbi.14252](https://doi.org/10.1111/pbi.14252)

9.	L. P. Hamel, F. Poirier-Gravel, M. Paré, R. Tardif, M. A. Comeau, P. O. Lavoie, A. Langlois, M. C. Goulet, D. Michaud, M. A. D’Aoust. Molecular changes in agroinfiltrated leaves of *Nicotiana benthamiana* expressing suppressor of silencing P19 and coronavirus-like particles. Plant Biotechnol J. 2026;24:99–130. doi:[10.1111/pbi.70075](https://doi.org/10.1111/pbi.70075)

10.	T. Sang, Z. Zhang, G. Liu, P. Wang. Navigating the landscape of plant proteomics. Journal of integrative plant biology. 2025;67:740–61. doi:[10.1111/jipb.13841](https://doi.org/10.1111/jipb.13841)

11.	Y. Xiao, S. L. Zhang, H. Yan, X. L. Geng, Y. W. Wang, X. Xu, M. Y. Wang, H. H. Zhang, B. C. Huang, W. Q. Pang, et al. The high immunity induced by the virus-like particles of foot-and-mouth disease virus serotype o. Frontiers in Veterinary Science. 2021;8. doi:[10.3389/fvets.2021.633706](https://doi.org/10.3389/fvets.2021.633706)

12.	X. H. Yao, A. Taheri, H. Liu, Y. J. Zhang, L. Li, J. Shao, K. Wu, Q. Miao, W. Z. He, X. Y. Hu, et al. Improvement and application of vacuum-infiltration system in tomato. Horticulture Research. 2024;11. doi:[10.1093/hr/uhae197](https://doi.org/10.1093/hr/uhae197)

13.	Q. Li, S. Wang, J. Wang, L. Chen, W. Liu, Z. Li, J. Xu, Z. Deng, Y. Zhou. Mechanism of phloretin accumulation in Malus hupehensis grown at high altitudes: evidence from quantitative 4D proteomics. J Agric Food Chem. 2024;72:19526–36. doi:10.1021/acs.jafc.4c04487

14.	J. Wang, Q. Zhang, J. Tung, X. Zhang, D. Liu, Y. Deng, Z. Tian, H. Chen, T. Wang, W. Yin, et al. High-quality assembled and annotated genomes of *Nicotiana tabacum* and *Nicotiana benthamiana* reveal chromosome evolution and changes in defense arsenals. Mol Plant. 2024;17:423–37. doi:[10.1016/j.molp.2024.01.008](https://doi.org/10.1016/j.molp.2024.01.008)

15.	J. Kourelis, F. Kaschani, F. M. Grosse-Holz, F. Homma, M. Kaiser, R. A. L. van der Hoorn. A homology-guided, genome-based proteome for improved proteomics in the alloploid *Nicotiana benthamiana*. BMC Genomics. 2019;20:722. doi:[10.1186/s12864-019-6058-6](https://doi.org/10.1186/s12864-019-6058-6)

16.	K. J. Livak, T. D. Schmittgen. Analysis of relative gene expression data using real-time quantitative PCR and the 2−ΔΔCT method. Methods. 2001;25:402–8. doi:10.1006/meth.2001.1262

17.	S. Bilotta, G. Éthier, A. C. Laliberté, M. C. Goulet, M. Martel, D. Michaud, S. Pepin. Synergetic light and cytokinin treatments mitigate the recombinant protein yield depression induced by high-density cultivation of hydroponically-grown *Nicotiana benthamiana*. Biotechnology and Bioengineering. 2024;121:3319–28. doi:[10.1002/bit.28781](https://doi.org/10.1002/bit.28781)

18.	C. Zipfel, G. Kunze, D. Chinchilla, A. Caniard, J. D. Jones, T. Boller, G. Felix. Perception of the bacterial PAMP EF-tu by the receptor EFR restricts agrobacterium-mediated transformation. Cell. 2006;125:749–60. doi:[10.1016/j.cell.2006.03.037](https://doi.org/10.1016/j.cell.2006.03.037)

19.	N. Prudhomme, R. Pastora, S. Thomson, E. Zheng, A. Sproule, J. R. Krieger, J. P. Murphy, D. P. Overy, D. Cossar, M. D. McLean, et al. Bacterial growth-mediated systems remodelling of *Nicotiana benthamiana* defines unique signatures of target protein production in molecular pharming. Plant Biotechnol J. 2024;22:2248–66. doi:[10.1111/pbi.14342](https://doi.org/10.1111/pbi.14342)

20.	F. Grosse-Holz, S. Kelly, S. Blaskowski, F. Kaschani, M. Kaiser, R. A. L. van der Hoorn. The transcriptome, extracellular proteome and active secretome of agroinfiltrated *Nicotiana benthamiana* uncover a large, diverse protease repertoire. Plant Biotechnol J. 2018;16:1068–84. doi:[10.1111/pbi.12852](https://doi.org/10.1111/pbi.12852)

21.	N. D. Rawlings, M. Waller, A. J. Barrett, A. Bateman. MEROPS: The database of proteolytic enzymes, their substrates and inhibitors. Nucleic Acids Res. 2014;42:D503–509. doi:[10.1093/nar/gkt953](https://doi.org/10.1093/nar/gkt953)

22.	P. V. Jutras, F. Grosse-Holz, F. Kaschani, M. Kaiser, D. Michaud, R. A. L. van der Hoorn. Activity-based proteomics reveals nine target proteases for the recombinant protein-stabilizing inhibitor SlCYS8 in *Nicotiana benthamiana*. Plant Biotechnol J. 2019;17:1670–8. doi:[10.1111/pbi.13092](https://doi.org/10.1111/pbi.13092)

23.	M. Niemer, U. Mehofer, M. Verdianz, A. Porodko, P. Schähs, D. Kracher, B. Lenarcic, M. Novinec, L. Mach. *Nicotiana benthamiana* cathepsin b displays distinct enzymatic features which differ from its human relative and aleurain-like protease. Biochimie. 2016;122:119–25. doi:[10.1016/j.biochi.2015.06.017](https://doi.org/10.1016/j.biochi.2015.06.017)

24.	N. Hatsugai, M. Kuroyanagi, K. Yamada, T. Meshi, S. Tsuda, M. Kondo, M. Nishimura, I. Hara-Nishimura. A plant vacuolar protease, VPE, mediates virus-induced hypersensitive cell death. Science. 2004;305:855–8. doi:[10.1126/science.1099859](https://doi.org/10.1126/science.1099859)

25.	E. L. Thomas. Protein processing in *Nicotiana benthamiana* by vacuolar processing enzymes and other proteases [PhD thesis]. University of Oxford; 2020.

26.	K. Beritza, E. C. Watts, R. A. L. van der Hoorn. Improving transient protein expression in agroinfiltrated *Nicotiana benthamiana*. New Phytol. 2024;243:846–50. doi:[10.1111/nph.19894](https://doi.org/10.1111/nph.19894)
