# Supplementary material for: 4D-DIA proteomics reveals distinct proteolytic landscapes induced by mechanical stress, Agrobacterium, and a viral capsid precursor
Source: PLoS One. 2026 Sep 11;21(9):e0356905. doi: 10.1371/journal.pone.0356905 (PMC13567786; doi:10.1371/journal.pone.0356905)
Supplement: Supplementary file 10003 [file pone.0356905.s006.pdf]

Manuscript

Click here to access/download;Manuscript;Tang_4D-DIA.pdf

4D-DIA Proteomics Reveals Distinct Proteolytic Landscapes Induced by Mechanical Stress, *Agrobacterium*, and Complex Viral Particles

You Tang[1◕], Xiaoping Qiu[2◕], Xiaorui Ma[2], Xinli Ye[2], Yilin Mao[2], Qiwei Yang[2], Yike Chen[2], Yiming Sun [2*], Lijun Zhu[1*],

1Chongqing Key Laboratory of Scientific Utilization of Tobacco Resources, Chongqing, China.
2 School of Life Science, Southwest University, Chongqing, China.

◕These authors contributed equally to this work.
* correspondingauthor: s8355@swu.edu.cn (Y.S.)
zhulj7802@163.com (L.Z.)

Abstract

Nicotiana benthamiana is a widely used platform for plant molecular farming, yet recombinant protein yields are frequently compromised by the host's innate defense mechanisms, particularly proteolytic degradation. While the general effects of Agroinfiltration are known, the distinct contributions of mechanical injury, bacterial perception, and product-specific stress remain poorly resolved. Here we utilized high-depth 4D-DIA proteomics to dissect the host response across three dimensions: physical stress (buffer infiltration), pathogen-associated stress (*Agrobacterium*), and product-associated stress (GFP vs. FMDV VLPs). We demonstrate that buffer infiltration is not a neutral event but an independent inducer of cell wall remodeling and oxidative stress. By filtering out these background effects, we defined a core Agrobacterium-responsive proteome characterized by a growth-defense trade-off. Furthermore, we expanded the known protease repertoire of *N. benthamiana* to 1,505 enzymes through improved genomic annotation. We found that the expression of virus-like particles (P1_2A) triggered a significantly more aggressive proteolytic environment compared to soluble GFP, specifically upregulating subtilases and cysteine proteases. These findings suggest that host proteolytic responses are cargo-dependent, necessitating tailored engineering strategies for the production of complex biopharmaceuticals.

Author summary

Plants are increasingly used as biofactories to produce vaccines and therapeutics. However, a major bottleneck is that plant cells often degrade these valuable recombinant proteins before harvest. While it is widely assumed that this degradation is primarily

triggered by the *Agrobacterium* used for gene delivery, we made an unexpected discovery. Using high-resolution 4D-DIA proteomics, we show that the simple act of buffer infiltration—a routine step in the process—already primes the plant's defense system, triggering early wound and oxidative stress responses that were previously attributed solely to the bacteria. More importantly, we found that complex viral antigens (forming virus-like particles) are attacked far more aggressively by the plant's proteolytic machinery than simple reporter proteins like GFP. This cargo-dependent response means that a "one-size-fits-all" protection strategy is ineffective. Our findings provide a roadmap for designing product-specific engineering strategies, ultimately making plant-based medicine production more practical and affordable.

Introduction

Plant molecular farming (PMF) is defined as the use of plant-based platforms to produce valuable proteins or metabolites that are used in the biopharmaceutical, nutraceutical or cosmetic industries, and more. *Nicotiana benthamiana* is the preferred host due to its susceptibility to transfection, rapid growth, and high biomass generation [1]. Despite these advantages, the accumulation of exogenously expressed proteins is often limited by the plant's physiological transition from a growth state to a defense state upon agroinfiltration [2]. This defense response creates a proteolytic environment in the apoplast and intracellular compartments, leading to product degradation and reduced yields [3] [4]. Although strategies such as co-expression of protease inhibitors have mitigated some losses [5] [6], current approaches often rely on a generalized understanding of stress rather than addressing specific degradation pathways. The stress imposed during transient expression is cumulative and multi-layered. The infiltration process itself applies significant mechanical pressure to leaf tissue, potentially triggering wound responses that are distinct from the immune responses elicited by *Agrobacterium tumefaciens* pathogen-associated molecular patterns (PAMPs) [7]. Furthermore, the physicochemical properties of the recombinant product influence the magnitude of cellular stress. Complex proteins, such as virus-like particles (VLPs), require extensive folding and assembly machinery within the endoplasmic reticulum (ER). Recent transcriptomic studies suggest that VLP expression triggers the unfolded protein response (UPR) and immune signaling more intensely than simple soluble proteins like Green Fluorescent Protein (GFP) [8] [9]. However, proteomic evidence distinguishing these layers of stress remains limited. To address these complexities, we employed 4D-DIA (Data-Independent Acquisition) mass spectrometry, which incorporates ion mobility separation to achieve superior sensitivity and proteome coverage compared to traditional methods [10]. We designed an experiment to systematically decouple the sources of host stress by comparing non-infiltrated wild-type plants (WT), buffer-infiltrated controls (mechanical stress), empty vector *Agrobacterium* (bacterial stress), and *Agrobacterium* expressing either GFP or the Foot-and-Mouth Disease Virus antigen P1_2A (product-specific stress) [11]. This study provides a high-resolution map of the *N. benthamiana* proteome dynamics, revealing that the host proteolytic landscape is actively reshaped by the specific nature of the recombinant cargo. We aimed to: (1) determine whether buffer infiltration alone induces a distinct proteomic signature separate from the Agrobacterium-mediated immune response; (2) define the core Agrobacterium-responsive proteome independent of cargo effects; and (3) test the hypothesis that host proteolytic responses are cargo-dependent, with complex viral antigens triggering a more aggressive protease induction than simple soluble proteins.

Materials and methods

Plant Materials and Agroinfiltration

Wild-type *Nicotiana benthamiana* plants were grown in chambers at 25 °C under a 16 h light/8 h dark photoperiod. *Agrobacterium tumefaciens* strain GV3101 harboring binary vectors for GFP, FMDV P1_2A [11], or an empty vector control was cultured in LB medium. Cells were resuspended in infiltration buffer (10 mM MES, 10 mM MgCl, 100 µM acetosyringone) and adjusted to an OD600 of 0.6. Infiltration was performed using a vacuum-infiltration system on leaves of 5-week-old plants according to Yao et al. [12].

Proteomic Analysis (4D-DIA)

Leaf samples were harvested at 1, 4, and 7 dpi, flash-frozen in liquid nitrogen, and ground to a fine powder. Total proteins were extracted using a phenol-based method. Protein concentration was determined using a BCA assay, and protein integrity was verified by sodium dodecyl sulfate-polyacrylamide gel electrophoresis (SDS-PAGE). Samples were shipped on dry ice to Majorbio Biomedical Technology Co., Ltd. (Shanghai, China) for LC-MS/MS analysis. Proteins were digested with trypsin (enzyme:substrate ratio of 1:50, w/w) at 37 °C for 16 h. The resulting peptides were desalted and separated on a nanoElute UHPLC system (Bruker) equipped with a C18 analytical column (75 m × 25 cm, 1.6 m particle size) at a flow rate of 300 nL/min over a 60-min linear gradient. Mobile phase A consisted of 98% water, 2% acetonitrile, and 0.1% formic acid; mobile phase B consisted of 80% acetonitrile, 20% water, and 0.1% formic acid. Data acquisition was performed on a timsTOF Pro mass spectrometer (Bruker Daltonics) operating in 4D-DIA mode, which leverages trapped ion mobility spectrometry (TIMS) to separate ions based on their collisional cross-section, providing an additional dimension of separation beyond retention time, m/z, and intensity [10]. The MS1 scan range was set to m/z 100-1700, and the TIMS ramp time was 100 ms with an ion mobility range (1/K0) of 0.6-1.6 V·s/cm^2.

Database search and quantification

Raw data were processed using DIA-NN (version 1.8.1) with a library-free search strategy. A spectral library was predicted in silico from the *N. benthamiana* proteome database (HZ version 1) [13] using the built-in deep learning module of DIA-NN. Trypsin/P was specified as the digestion enzyme with up to one missed cleavage allowed. Carbamidomethylation of cysteine was set as a fixed modification, and oxidation of methionine and N-terminal acetylation were set as variable modifications. The precursor mass tolerance and fragment mass tolerance were determined automatically by DIA-NN. The false discovery rate (FDR) was controlled at ¡ 1% at both the precursor ion and protein group levels using a target-decoy strategy. Protein quantification was performed using the MaxLFQ algorithm integrated in DIA-NN, with match-between-runs enabled to maximize identification transfer across samples. Peptide-spectrum matches (PSMs) with a posterior error probability (PEP) < 0.01 were retained. A total of 88,895 PSMs and 72,464 unique peptide sequences were identified and used for protein-level inference, yielding 10,597 quantified protein groups across all 45 samples.

Bioinformatics analysis

A reference database containing specific Pfam domain IDs associated with proteases was constructed, referencing established classifications [14]. The protein sequences of the latest *N. benthamiana* genome HZ version 1 [13] were scanned for functional domains

using hmmer (version 3.3) with default E-value thresholds (sequence E-value < 10, domain E-value < 10). Proteins were categorized by matching their identified Pfam domains against the reference dataset (Table S3). Prior to differential expression analysis, an abundance-based pre-filtering step was applied. The first quartile (Q1) of protein abundances across all control samples (WT and buffer groups at all time points) was calculated as a baseline threshold. Only proteins with a maximum abundance exceeding this Q1 value in at least one control sample were retained for subsequent identification of differentially expressed proteins (DEPs). Differentially expressed proteins (DEPs) were identified using pairwise Student's t-tests between treatment groups and controls. Proteins with an absolute fold change > 1.5 and a Benjamini-Hochberg adjusted P-value (FDR) < 0.05 were defined as significantly differentially expressed. The experimental design comprised five treatment groups (WT, Buffer, GV3101, GFP, and P1_2A) sampled at three time points (1, 4, and 7 dpi), with three independent biological replicates per condition, yielding a total of 45 samples. To elucidate the biological functions of the differentially expressed proteins (DEPs) in each comparison, comprehensive functional enrichment analyses were performed encompassing Gene Ontology (GO) terms, KOG classifications, KEGG pathways, and protein domains. A hypergeometric test was employed to calculate the statistical significance (P-value), identifying specific functional categories that were significantly overrepresented among the DEPs relative to the entire identified proteome as the background.

Quantitative real-time PCR analysis

Total RNA was isolated from *N. benthamiana* leaves using TRIzol Reagent (Invitrogen) and subsequently treated with DNase I to eliminate genomic DNA contamination. First-strand cDNA was synthesized employing the HiScript® II Q RT SuperMix (+gDNA wiper) kit (Vazyme). Quantitative real-time PCR (qRT-PCR) was performed in technical triplicates on a CFX Connect Real-Time PCR Detection System (Bio-Rad) utilizing SYBR Green chemistry. The thermocycling parameters consisted of 40 cycles at 95 °C for 10 s and 60 °C for 30 s, with fluorescence signals acquired at the end of each cycle. Relative gene expression levels were determined via the $2\hat{(}Ct)$ method, utilizing -actin as the reference gene. Primer sequences are listed in Table S4. All analyses were conducted with three biological replicates.

Results

Deep Proteome Coverage via 4D-DIA Captures Subtle Host Dynamics

We utilized the 4D-DIA platform to analyze the temporal dynamics of the *N. benthamiana* proteome at 1, 4, and 7 days post-infiltration (dpi). The experimental design included five treatment groups to isolate specific stress factors (Fig 1A). A total of 88895 peptide-spectrum matches (PSMs) and 72464 unique peptide sequences were identified from the raw data and were employed for protein mapping. As a result, 9495 protein groups were identified across three independent biological replicates and subsequently quantified using the MS1 intensity-based label-free quantification (LFQ) method (Table S1). Pearson correlation analysis confirmed high reproducibility across all 45 proteomes, with average correlation coefficients exceeding 0.95 within each treatment group (Fig 1B). UMAP dimensionality reduction of the global proteome revealed a clear topological separation between agroinfiltrated and non-infiltrated samples: WT and buffer controls formed a distinct cluster from the GV3101, GFP, and

P1_2A groups, and this divergence intensified progressively from 1 to 7 dpi (Fig 1C). This global trajectory demonstrates that Agrobacterium-mediated transformation fundamentally reshapes the host proteome in a time-dependent manner. Notably, the buffer-infiltrated group showed clear separation from the non-infiltrated WT group as early as 1 dpi (Fig 1D). This separation persisted throughout 1 and 4 dpi, indicating that the physical act of infiltration induces a proteomic shift.

Fig 1. Experimental design and global proteomic landscape of *N. benthamiana* responses to agroinfiltration. (A) Experimental workflow. *N. benthamiana* leaves were subjected to five treatments: untreated wild type (WT), buffer control, empty *Agrobacterium* (GV3101), and *Agrobacterium* expressing GFP or FMDV antigen P1_2A. Samples were harvested at 1, 4, and 7 days post-infiltration (dpi) and analyzed via 4D-DIA LC-MS/MS (n=3 biological replicates). (B) Pearson correlation heatmap of all 45 proteomes. (C) UMAP visualization of the global proteomic trajectory, highlighting temporal shifts and distinct clustering between non-infiltrated controls (WT/buffer) and *Agrobacterium*-infiltrated groups. (D) PCA plots at individual time points (1, 4, and 7 dpi).

Buffer Infiltration Triggers Early Defense and Wound Responses

To understand the "background noise" in molecular farming experiments, we analyzed differentially expressed proteins (DEPs) between the Buffer and WT groups. The volcano plots reveal that the "mock" treatment is biologically active, inducing significant protein changes as early as 1 dpi (Fig 2A, Table S2). Functional enrichment analysis (GO and KEGG) indicated a significant upregulation of proteins involved in phenylpropanoid biosynthesis, MAPK signaling, and oxidoreductase activity (Fig 2B). Specifically, terms such as "cell wall biogenesis," "apoplast," and "hydrogen peroxide catabolic process" were enriched (Fig 2C). This molecular signature aligns with a classic wound response, where mechanical damage from high-pressure infiltration triggers the reinforcement of physical barriers and a burst of reactive oxygen species (ROS) [7]. These data confirm that buffer infiltration establishes a high-stress baseline environment characterized by oxidative stress and cell wall remodeling, independent of any biological agent.

Fig 2. Time-course proteomic profiling and functional enrichment in response to buffer infiltration. (A) Volcano plots of differentially expressed proteins (DEPs) between buffer-infiltrated and untreated WT leaves at 1, 4, and 7 days post-infiltration (dpi). Red and blue dots indicate significantly up- and down-regulated proteins, respectively (fold change > 1.5, $P < 0.05$). (B) KEGG pathway enrichment analysis of the up- and down-regulated DEPs at 1, 4, and 7 dpi. (C) Gene Ontology (GO) enrichment analysis of biological processes and molecular functions for the up- and down-regulated DEPs at 1, 4, and 7 dpi.

The Core Agrobacterium-Responsive Proteome

Having established that buffer infiltration alone constitutes a significant abiotic stressor, we next sought to identify the proteomic changes attributable specifically to *Agrobacterium*. To isolate the host response specifically attributable to *Agrobacterium* infection, we identified the intersection of DEPs found in all bacteria-treated groups (GV3101, GFP, and P1_2A) relative to the Buffer control (Fig 3A, Table S2). This approach filters out mechanical stress, leaving a "core Agrobacterium-responsive proteome." This core response was characterized by extensive metabolic reprogramming consistent with the growth-defense trade-off hypothesis [15]. We observed a universal downregulation of proteins associated with photosynthesis (e.g., "photosynthesis, light harvesting," chlorophyll A-B binding proteins, and Rubisco small subunits) and carbon

metabolism across all time points (Fig 3B, 3C). This photosynthetic suppression mirrors the transcriptomic findings of Grosse-Holz et al. [16], who reported that chlorophyll A-B binding protein transcripts and carbon fixation genes were among the earliest to decline upon agroinfiltration. Our data confirm that this suppression is sustained at the protein level through at least 7 dpi, extending beyond the 5 dpi window examined in that study. Conversely, proteins involved in protein processing in the endoplasmic reticulum (ER), ribosome biogenesis, and vesicle trafficking were significantly upregulated (Fig 3B). The upregulation of ER protein processing components is consistent with the increased secretory burden imposed by *Agrobacterium* T-DNA-mediated gene expression, and aligns with Prudhomme et al. [17], who reported enrichment of ER-associated quality control proteins in their multi-omics analysis of agroinfiltrated *N. benthamiana*. KEGG pathway analysis further confirmed this metabolic shift, revealing the downregulation of photosynthesis-antenna proteins and carbon fixation pathways, while plant-pathogen interaction, MAPK signaling, and protein processing in the ER were enriched among the upregulated core proteins (Fig 3C). The enrichment of plant-pathogen interaction pathways is consistent with PAMP perception via pattern recognition receptors such as EFR, which detects the bacterial elongation factor EF-Tu [18]. At the metabolic level, Prudhomme et al. [17] demonstrated that the jasmonic acid (JA) precursor 12-OPDA and six fatty acid -oxidation enzymes accumulated in agroinfiltrated tissue, connecting PAMP-triggered immunity to hormonal defense signaling. Notably, the extracellular proteome analysis by Grosse-Holz et al. [16] showed that hydrolytic defense proteins, including chitinases (GH18), PR2 glucanases (GH17), and xylanase inhibitors, accumulated in the apoplast by 2 dpi, while papain-like cysteine proteases (PLCPs, family C01) did not increase proportionally in the extracellular fraction. In contrast, our total cellular proteome data captured the intracellular accumulation of these PLCPs, suggesting that many PLCPs induced during agroinfiltration are retained in vacuolar or ER-associated compartments rather than being secreted. The number of core DEPs increased from 1 to 7 dpi, indicating a progressive amplification of the host defense response over time. This temporal escalation is consistent with the pattern reported by Grosse-Holz et al. [16], in which the number of differentially abundant extracellular proteins expanded from 2 to 10 dpi.

Fig 3. Identification and functional enrichment of the core Agrobacterium-responsive proteome. (A) Venn diagrams displaying the overlap of DEPs among GV3101, GFP, and P1_2A treatments relative to buffer control at 1, 4, and 7 dpi. (B) Gene Ontology (GO) enrichment analysis of the up- and down-regulated core DEPs. (C) KEGG pathway enrichment analysis.

Expanded Protease Landscape and the Core Proteolytic Response

Given that proteolytic degradation is a primary barrier to recombinant protein accumulation, we next focused our analysis on the protease component of these responses. Proteases are the primary bottleneck in molecular farming. Leveraging the latest *N. benthamiana* genome assembly [14] and our deep proteomic data, we annotated 1,505 putative proteases, significantly expanding upon the 1,243 proteases previously reported [16]. These were classified into catalytic families based on MEROPS nomenclature [19], with serine (Ser), cysteine (Cys), and aspartic (Asp) proteases dominating the landscape (Fig 4A, Table S3). Hierarchical clustering showed that a large subset of these proteases is upregulated upon agroinfiltration regardless of the cargo (Fig 4B). We identified specific enzymes, such as the aspartic protease NbALP1, the cysteine protease NbCTB2, and the subtilase NbSBT6, as part of the core proteolytic response (Fig 4C). These enzymes showed consistent upregulation in

GV3101, GFP, and P1_2A groups compared to buffer controls. NbALP1 and NbCTB2 are known to be involved in the degradation of recombinant proteins in the apoplast [20] [21].

Fig 4. The expanded protease landscape of *N. benthamiana* and the core proteolytic response. (A) Classification of the 1,505 proteases identified in this study into MEROPS catalytic classes (Serine, Cysteine, Aspartic, Metallo, Threonine). (B) Hierarchical clustering of protease expression profiles across all treatments. Red indicates upregulation; blue indicates downregulation. (C) Expression profiles of representative "Core" proteases including NbALP1 (Aspartic), NbCTB2 (Cysteine), NbSBT6 (Serine), and NbVPE1b (Cysteine).

P1_2A Triggers a Product-Specific Proteolytic Storm

While the above analyses identified a common proteolytic response to *Agrobacterium*, a key question for molecular farming is whether the nature of the recombinant cargo further shapes the host response. A direct comparison between the GFP and P1_2A expression groups revealed marked differences in the host response. P1_2A, a viral capsid precursor that self-assembles into VLPs, induced a significantly higher number of DEPs compared to the soluble GFP reporter, particularly at 4 dpi (Fig 5B, Table S2). Functional enrichment analysis of the P1_2A-specific response highlighted pathways related to cytoskeleton organization ("microtubule-based process"), nuclear pore complex, and chromatin remodeling (Fig 5A). This likely reflects the complex cellular requirements for VLP assembly and trafficking [8]. Most critically, P1_2A expression triggered the specific and intense upregulation of a distinct subset of proteases (Fig 5D). Time-course analysis of recombinant protein accumulation revealed strikingly different dynamics between the two cargoes: GFP protein intensity peaked sharply at 4 dpi before declining precipitously by 7 dpi, whereas P1_2A protein levels remained consistently low throughout the time course (Fig 5B). A similar trend was observed for relative expression levels, with both recombinant proteins approaching baseline by 7 dpi, suggesting that host defense mechanisms ultimately suppress heterologous protein accumulation regardless of cargo identity (Fig 5C). Importantly, while GFP achieved substantially higher peak accumulation, the P1_2A group exhibited a more sustained and intense stress-related proteome. The heatmap of cargo-specific differentially expressed proteases revealed that 10 proteases spanning three catalytic classes—aspartic (Asp), cysteine (Cys), and serine (Ser)—were significantly differentially regulated between GFP and P1_2A at 4 dpi (Fig 5D). Specific proteases, including NbSBT9 (serine), NbRD21B (cysteine), and NbVPE1b (Vacuolar Processing Enzyme, cysteine), were hyper-induced in the P1_2A group, along with previously uncharacterized proteases Nbe16g06900.1 (Asp), Nbe19g17120.1 (Cys), and Nbe14g08810.1 (Cys). This suggests that the host cell perceives the structural complexity of P1_2A as a specific stressor, potentially exacerbating ER stress and activating UPR-associated degradation pathways involving VPEs [22] [23].

qRT-PCR Validation of Cargo-Specific Protease Induction

To validate the proteomic findings at the transcript level, we performed quantitative real-time PCR (qRT-PCR) analysis on ten selected protease genes that were differentially expressed in the P1_2A group (Fig 5E). The majority of the tested proteases, including NbSBT9 (2.29-fold), NbRD21B (2.53-fold, $P = 0.049$), NbALP1 (2.84-fold, $P = 0.014$), and Nbe16g06900.1 (2.18-fold), showed elevated transcript levels in both the GFP and P1_2A groups relative to the GV3101 empty vector control, confirming that these proteases are induced as part of the general Agrobacterium-responsive program. NbRD21B and Nbe16g06900.1 exhibited

statistically significant upregulation in the P1_2A group compared to GV3101 ($P = 0.006$ and $P = 0.003$, respectively). Notably, however, the transcript-level differences between P1_2A and GFP groups did not reach statistical significance for any of the tested genes ($P > 0.05$), despite clear differences at the protein level (Fig 5D). Two genes, Nbe19g17120.1 (1.60-fold, $P = 0.081$) and Nbe11g00200.1 (1.52-fold, $P = 0.092$), showed a trend toward higher expression in the P1_2A group but remained below the significance threshold. This discordance between protein and transcript abundance suggests that the cargo-specific protease accumulation observed in the P1_2A group is regulated, at least in part, at the post-transcriptional level, potentially involving differential mRNA stability, translational efficiency, or protein turnover rates.

Fig 5. Cargo-specific proteomic responses and qRT-PCR validation. (A) Gene Ontology (GO) functional enrichment analysis of differentially expressed proteins between P1_2A and GFP groups at 4 dpi. Node color indicates -log10(P-value) and node size indicates the protein ratio. (B) Time-course comparison of relative protein intensity for the recombinant proteins GFP and P1_2A at 1, 4, and 7 dpi (mean ± SD, n=3). (C) Relative expression levels of GFP and P1_2A over the infiltration time course, normalized to 1 dpi values. (D) Heatmap of cargo-specific differentially expressed proteases between GFP and P1_2A groups at 4 dpi. Proteases are classified by catalytic type: Asp (aspartic), Cys (cysteine), and Ser (serine). Red indicates upregulation; blue indicates downregulation. (E) qRT-PCR validation of ten selected protease genes. Bar plots show relative expression levels in GV3101, GFP, and P1_2A groups at 4 dpi (mean ± SD, n=3). Asterisks indicate statistical significance (*P < 0.05, Student's t-test).

Discussion

Our study provides a high-resolution, temporally resolved proteomic atlas of the multi-layered host response during agroinfiltration of *Nicotiana benthamiana*, revealing that the widely assumed "neutral" controls in molecular farming are biologically active. The buffer infiltration alone induced significant changes in the proteome, including the upregulation of cell wall remodeling enzymes, MAPK signaling components, and oxidoreductases as early as 1 dpi (Fig 2). This finding is consistent with the "infiltration stress" concept described by Hamel [2] and the metabolic perturbations reported by Drapal et al. [7] in different *N. benthamiana* accessions. The activation of phenylpropanoid biosynthesis pathways in our buffer-infiltrated samples mirrors the wound-induced lignification response documented in other systems. Prudhomme et al. [17] similarly noted that bacterial growth alone reshapes the *N. benthamiana* metabolome, but our data extend this by showing that even the mechanical component of infiltration, without any bacterial presence, is sufficient to prime the defense transcriptome. This has practical implications: experiments using buffer-infiltrated controls as a "baseline" may systematically underestimate the total stress burden imposed on the plant, as these controls already represent a perturbed state. By subtracting the buffer infiltration background, we defined a core Agrobacterium-responsive proteome that was shared across all bacteria-treated groups regardless of the expressed cargo. This core response was dominated by a growth-defense trade-off, with photosynthetic proteins consistently downregulated and defense-related proteins upregulated (Fig 3). This pattern aligns closely with the transcriptomic findings of Grosse-Holz et al. [16], who reported a similar suppression of photosynthesis-related transcripts during agroinfiltration. Our proteomic data confirm that this trade-off is also manifest at the protein level and persists across the 1–7 dpi time window. The metabolic cost of mounting this defense was recently shown to be partially reversible through light and cytokinin co-treatments [15], which underlines the need to understand these basal responses for yield optimization. The upregulation of

ER-associated protein processing and vesicle trafficking components in the core response suggests that PAMP recognition by receptors such as EFR [18] triggers a sustained secretory pathway remodeling, which may compete with recombinant protein folding and secretion. Perhaps the most consequential finding for the molecular farming is the demonstration that host proteolytic responses are not uniform but cargo-dependent. The P1_2A viral capsid precursor induced a quantitatively and qualitatively distinct protease profile compared to soluble GFP (Fig 5D). The specific upregulation of subtilases (e.g., NbSBT9), papain-like cysteine proteases (e.g., NbRD21B), and vacuolar processing enzymes (e.g., NbVPE1b) in the P1_2A group points to the activation of both apoplastic and vacuolar degradation pathways. This is consistent with recent findings that coronavirus-like particles (CoVLPs) expressed in *N. benthamiana* trigger an intense unfolded protein response (UPR) and specific immune signaling cascades distinct from those induced by simpler proteins [8] [9]. Grosse-Holz et al. [16] identified approximately 1,243 proteases from their transcriptome and secretome analyses; our 4D-DIA approach expanded this repertoire to 1,505 enzymes, likely reflecting the improved sensitivity afforded by ion mobility separation and the use of an updated genome assembly. Importantly, our qRT-PCR data revealed that the cargo-specific differences in protease abundance are not fully reflected at the transcript level, as P1_2A vs. GFP comparisons did not reach statistical significance for any of the ten tested protease genes (Fig 5E). This discordance suggests that post-transcriptional regulation, such as differential mRNA stability or protein turnover, plays a significant role in shaping the cargo-specific proteolytic landscape. These findings argue against the ”one-size-fits-all” approach to protease inhibitor co-expression and instead support targeted strategies: for complex antigens like P1_2A, silencing or inhibiting specific SBTs and PLCPs [6] [24] may be more effective than broad-spectrum approaches. The 4D-DIA approach enabled the identification of 10,597 protein groups across all samples, achieving deeper proteome coverage than previous DDA-based studies of agroinfiltrated *N. benthamiana*. The incorporation of ion mobility as a fourth separation dimension enhanced peptide identification confidence and quantification accuracy, particularly for low-abundance regulatory proteins such as proteases and signaling kinases. The annotation of 1,505 putative proteases, representing a 2̃1% increase over the 1̃,243 reported by Grosse-Holz et al. [16], can be attributed to three factors: (i) the use of the updated HZ version 1 genome assembly [13], which provides more complete gene models; (ii) the superior sensitivity of 4D-DIA in detecting low-abundance proteins; and (iii) systematic hmmer-based domain scanning against the MEROPS/Pfam reference database with default E-value thresholds. However, some of these annotations remain putative and would benefit from experimental validation of proteolytic activity, for instance through activity-based protein profiling as described by Jutras et al. [20]. This study has several limitations. First, our analysis was conducted using a single *N. benthamiana* accession under controlled growth conditions; the generalizability of these findings to other accessions or field-grown plants remains to be established [7]. Second, we sampled at three time points (1, 4, and 7 dpi), which captures the major temporal dynamics but may miss early signaling events occurring within hours of infiltration. Third, we used a single *Agrobacterium* density (OD600 = 0.6); dose-dependent effects on the host proteome were not explored. Fourth, while our qRT-PCR validation targeted ten protease genes, confirmation of the broader proteomic findings would benefit from ~~orthogonal~~ approaches such as Western blotting or targeted proteomics (PRM/SRM). Finally, the mechanistic basis of the cargo-specific protease induction—whether driven by ER stress sensing, T-DNA integration efficiency, or direct protein–protein interactions—is not yet clear. Future studies combining genetic perturbation (e.g., CRISPR-mediated knockout of key SBTs and PLCPs) with time-resolved proteomics will be essential to translate these observations into actionable

strategies for improving recombinant protein yields in plant molecular farming.

Conclusion

In conclusion, this study provides three main insights. First, buffer infiltration is not a neutral control but actively primes wound and oxidative stress responses, confounding the interpretation of bacterial and product-specific effects. Second, the core Agrobacterium-responsive proteome is defined by a sustained growth-defense trade-off, explaining the metabolic cost of transient expression. Third, host proteolytic responses are cargo-dependent: the complex VLP antigen P1_2A elicits a distinct and more aggressive protease signature—enriching subtilases and cysteine proteases—regulated predominantly at the post-transcriptional level. These findings argue against broad-spectrum protease inhibitors and support precision-targeted strategies tailored to the target product.

Supporting information

S1 Table. Summary of proteomic data acquisition and identification.

S2 Table. Summary of differentially expressed proteins (DEPs) between Buffer and WT groups at 1, 4, and 7 days post-infiltration (dpi).

S3 Table. Annotation of putative proteases identified in *Nicotiana benthamiana* with MEROPS-based classification and Pfam domain information.

S4 Table. Primers used for RT-qPCR.

Acknowledgments

This research was funded by the Open Project of Chongqing Key Laboratory of Scientific Utilization of Tobacco Resources, grant number HX20230208; and the Chongqing College Student Innovation and Entrepreneurship Training Program, grant number S20241063378.

References

1. Bally J, Jung H, Mortimer C, Naim F, Philips JG, Hellens R, et al. The Rise and Rise of Nicotiana benthamiana: A Plant for All Reasons. Annu Rev Phytopathol. 2018;56:405-26. doi:10.1146/annurev-phyto-080417-050141.

2. Hamel L. Nicotiana benthamiana's Responses to Agroinfiltration, a Treasure Grove of New Avenues to Improve Protein Yields in Plant Molecular Farming. Plant Biotechnol J. 2026;24:5-17. doi:10.1111/pbi.70460.

3. Jutras PV, Dodds I, van der Hoorn RA. Proteases of Nicotiana benthamiana: an emerging battle for molecular farming. Curr Opin Biotechnol. 2020;61:60-5. doi:10.1016/j.copbio.2019.10.006.

4. Mandal MK, Ahvari H, Schillberg S, Schiermeyer A. Tackling Unwanted Proteolysis in Plant Production Hosts Used for Molecular Farming. Front Plant Sci. 2016;7. doi:10.3389/fpls.2016.00267.

5. Goulet C, Khalf M, Sainsbury F, D'Aoust MA, Michaud D. A protease activity-depleted environment for heterologous proteins migrating towards the leaf cell apoplast. Plant Biotechnol J. 2012;10:83-94. doi:10.1111/j.1467-7652.2011.00643.x.

6. Grosse-Holz F, Madeira L, Zahid MA, Songer M, Kourelis J, Fesenko M, et al. Three unrelated protease inhibitors enhance accumulation of pharmaceutical recombinant proteins in Nicotiana benthamiana. Plant Biotechnol J. 2018;16:1797-810. doi:10.1111/pbi.12916.

7. Drapal M, Enfissi EMA, Fraser PD. Metabolic effects of agro-infiltration on N. benthamiana accessions. Transgenic Research. 2021;30:303-15. doi:10.1007/s11248-021-00256-9.

8. Hamel LP, Comeau MA, Tardif R, Poirier-Gravel F, Paré M, Lavoie PO, et al. Heterologous expression of influenza haemagglutinin leads to early and transient activation of the unfolded protein response in Nicotiana benthamiana. Plant Biotechnol J. 2024;22:1146-63. doi:10.1111/pbi.14252.

9. Hamel LP, Poirier-Gravel F, Paré M, Tardif R, Comeau MA, Lavoie PO, et al. Molecular changes in agroinfiltrated leaves of Nicotiana benthamiana expressing suppressor of silencing P19 and coronavirus-like particles. Plant Biotechnol J. 2026;24:99-130. doi:10.1111/pbi.70075.

10. Sang T, Zhang Z, Liu G, Wang P. Navigating the landscape of plant proteomics. Journal of integrative plant biology. 2025;67:740-61. doi:10.1111/jipb.13841.

11. Xiao Y, Zhang SL, Yan H, Geng XL, Wang YW, Xu X, et al. The High Immunity Induced by the Virus-Like Particles of Foot-and-Mouth Disease Virus Serotype O. Frontiers in Veterinary Science. 2021;8. doi:10.3389/fvets.2021.633706.

12. Yao XH, Taheri A, Liu H, Zhang YJ, Li L, Shao J, et al. Improvement and application of vacuum-infiltration system in tomato. Horticulture Research. 2024;11. doi:10.1093/hr/uhae197.

13. Wang J, Zhang Q, Tung J, Zhang X, Liu D, Deng Y, et al. High-quality assembled and annotated genomes of Nicotiana tabacum and Nicotiana benthamiana reveal chromosome evolution and changes in defense arsenals. Mol Plant. 2024;17:423-37. doi:10.1016/j.molp.2024.01.008.

14. Kourelis J, Kaschani F, Grosse-Holz FM, Homma F, Kaiser M, van der Hoorn RAL. A homology-guided, genome-based proteome for improved proteomics in the alloploid Nicotiana benthamiana. BMC Genomics. 2019;20:722. doi:10.1186/s12864-019-6058-6.

15. Bilotta S, Éthier G, Laliberté AC, Goulet MC, Martel M, Michaud D, et al. Synergetic light and cytokinin treatments mitigate the recombinant protein yield depression induced by high-density cultivation of hydroponically-grown Nicotiana benthamiana. Biotechnology and Bioengineering. 2024;121:3319-28. doi:10.1002/bit.28781.

16. Grosse-Holz F, Kelly S, Blaskowski S, Kaschani F, Kaiser M, van der Hoorn RAL. The transcriptome, extracellular proteome and active secretome of agroinfiltrated Nicotiana benthamiana uncover a large, diverse protease repertoire. Plant Biotechnol J. 2018;16:1068-84. doi:10.1111/pbi.12852.

17. Prudhomme N, Pastora R, Thomson S, Zheng E, Sproule A, Krieger JR, et al. Bacterial growth-mediated systems remodelling of Nicotiana benthamiana defines unique signatures of target protein production in molecular pharming. Plant Biotechnol J. 2024;22:2248-66. doi:10.1111/pbi.14342.

18. Zipfel C, Kunze G, Chinchilla D, Caniard A, Jones JD, Boller T, et al. Perception of the bacterial PAMP EF-Tu by the receptor EFR restricts Agrobacterium-mediated transformation. Cell. 2006;125:749-60. doi:10.1016/j.cell.2006.03.037.

19. Rawlings ND, Waller M, Barrett AJ, Bateman A. MEROPS: the database of proteolytic enzymes, their substrates and inhibitors. Nucleic Acids Res. 2014;42:D503-9. doi:10.1093/nar/gkt953.

20. Jutras PV, Grosse-Holz F, Kaschani F, Kaiser M, Michaud D, van der Hoorn RAL. Activity-based proteomics reveals nine target proteases for the recombinant protein-stabilizing inhibitor SlCYS8 in Nicotiana benthamiana. Plant Biotechnol J. 2019;17:1670-8. doi:10.1111/pbi.13092.

21. Niemer M, Mehofer U, Verdianz M, Porodko A, Schähs P, Kracher D, et al. Nicotiana benthamiana cathepsin B displays distinct enzymatic features which differ from its human relative and aleurain-like protease. Biochimie. 2016;122:119-25. doi:10.1016/j.biochi.2015.06.017.

22. Hatsugai N, Kuroyanagi M, Yamada K, Meshi T, Tsuda S, Kondo M, et al. A plant vacuolar protease, VPE, mediates virus-induced hypersensitive cell death. Science. 2004;305:855-8. doi:10.1126/science.1099859.

23. Thomas EL. Protein Processing in Nicotiana Benthamiana by Vacuolar Processing Enzymes and Other Proteases. University of Oxford; 2020.

24. Beritza K, Watts EC, van der Hoorn RAL. Improving transient protein expression in agroinfiltrated Nicotiana benthamiana. New Phytol. 2024;243:846-50. doi:10.1111/nph.19894.

Fig 1

Click here to access/download;Figure;fig1.jpg

A IS NOT NEEDED

ALL SEEM OF LOW RESOLUTION

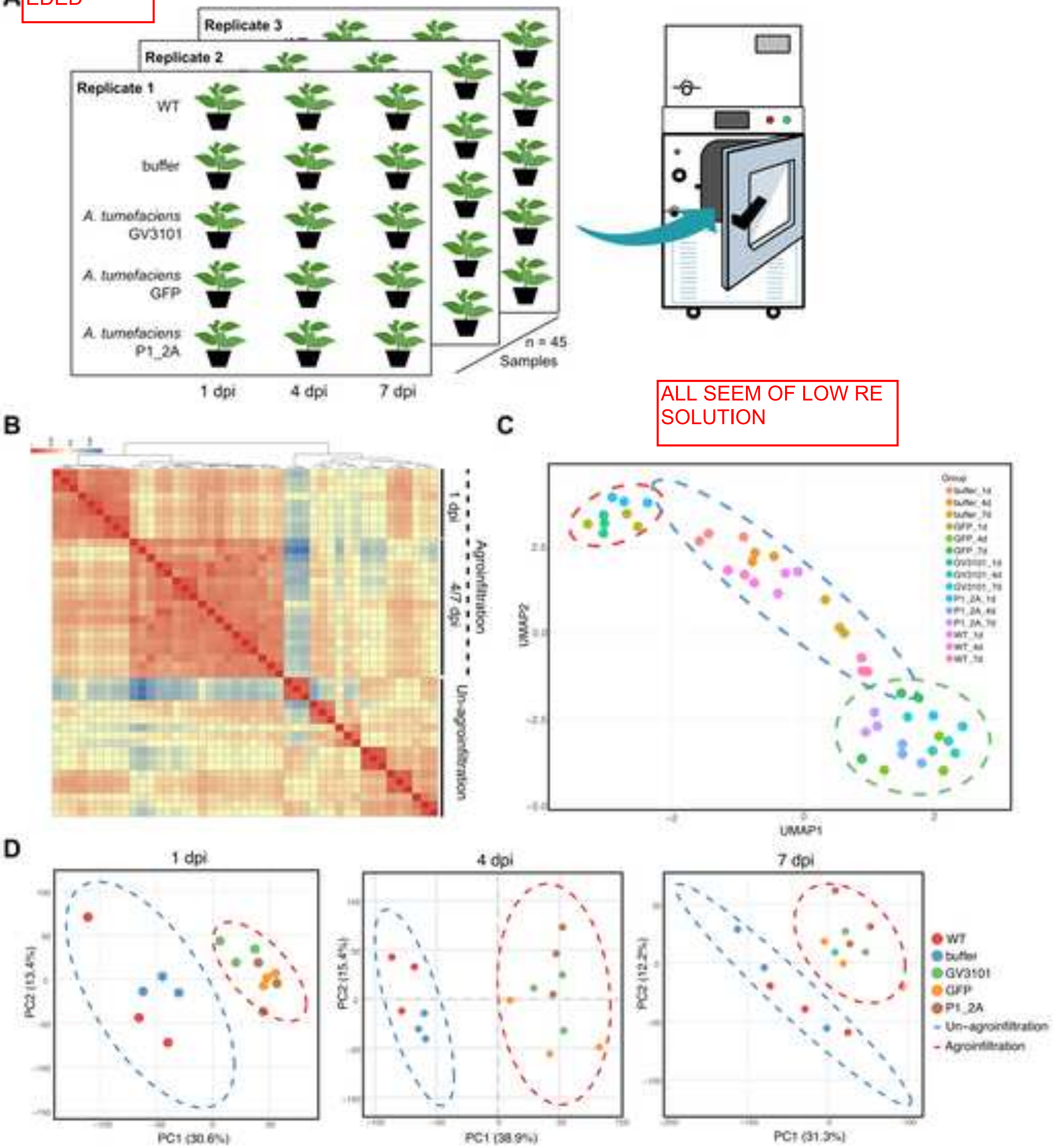

Fig 2

Click here to access/download;Figure;fig2.jpg

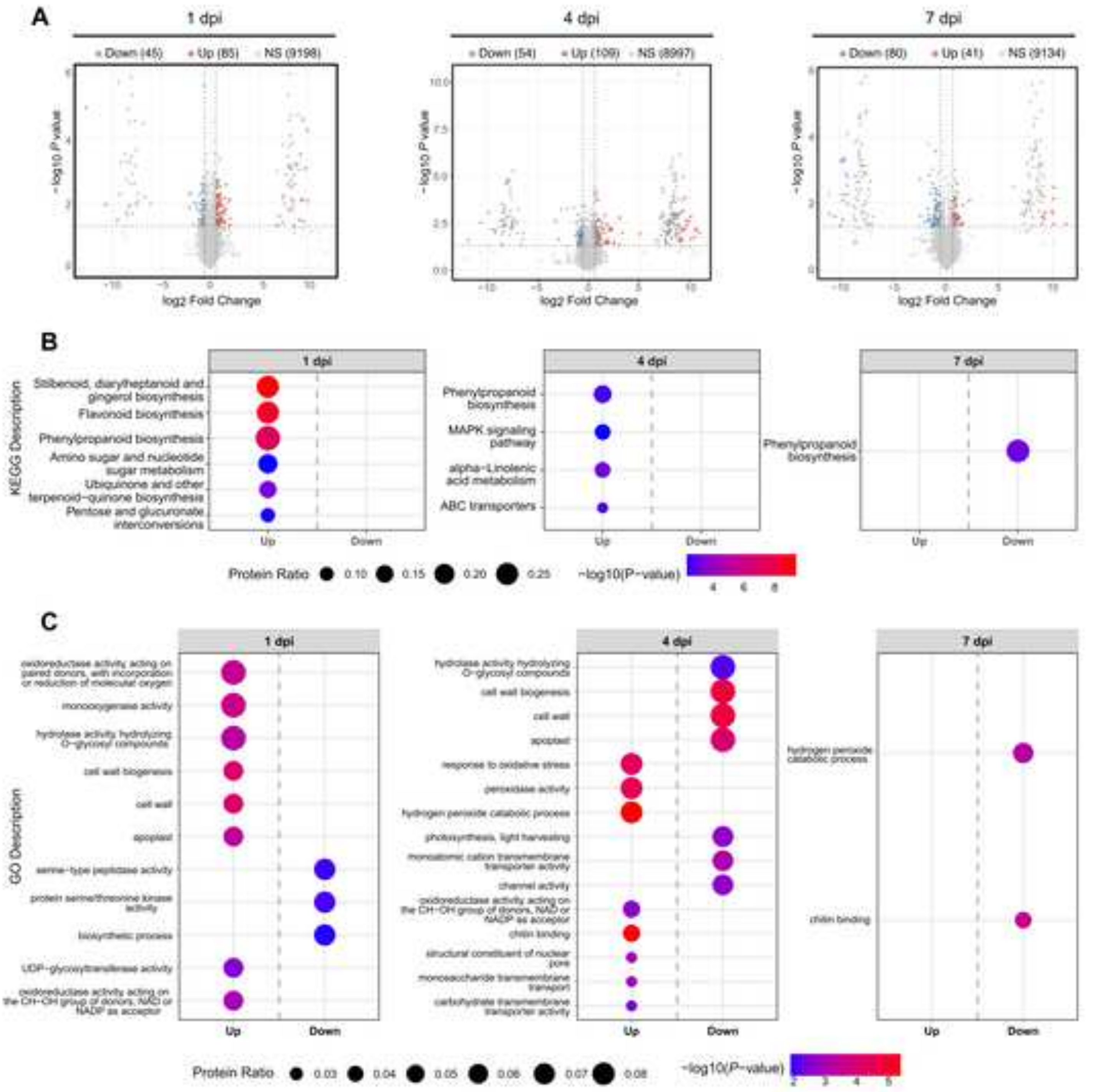

Fig 3

Click here to access/download;Figure;fig3.jpg

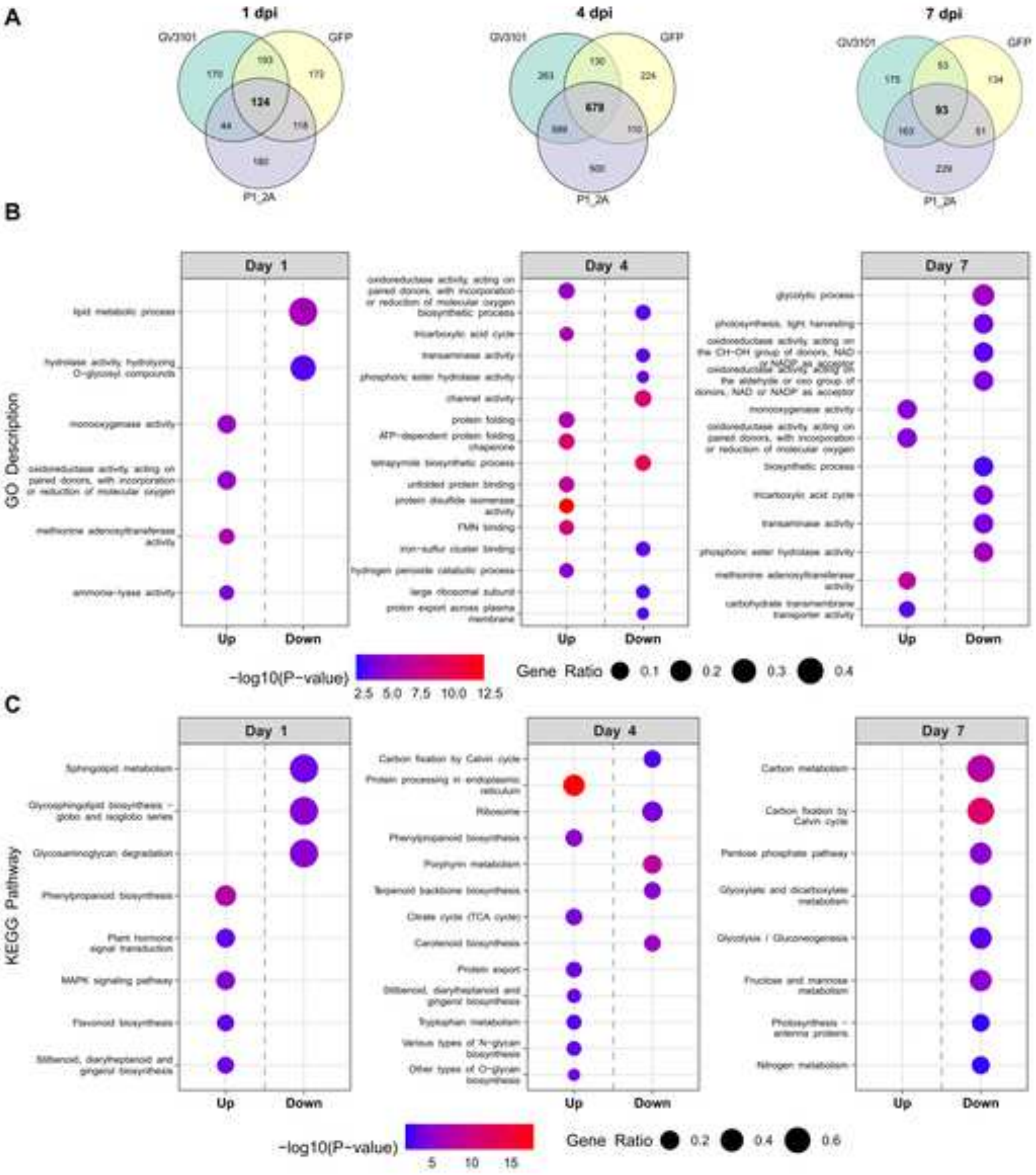

Fig 4

Click here to access/download;Figure;fig4.jpg

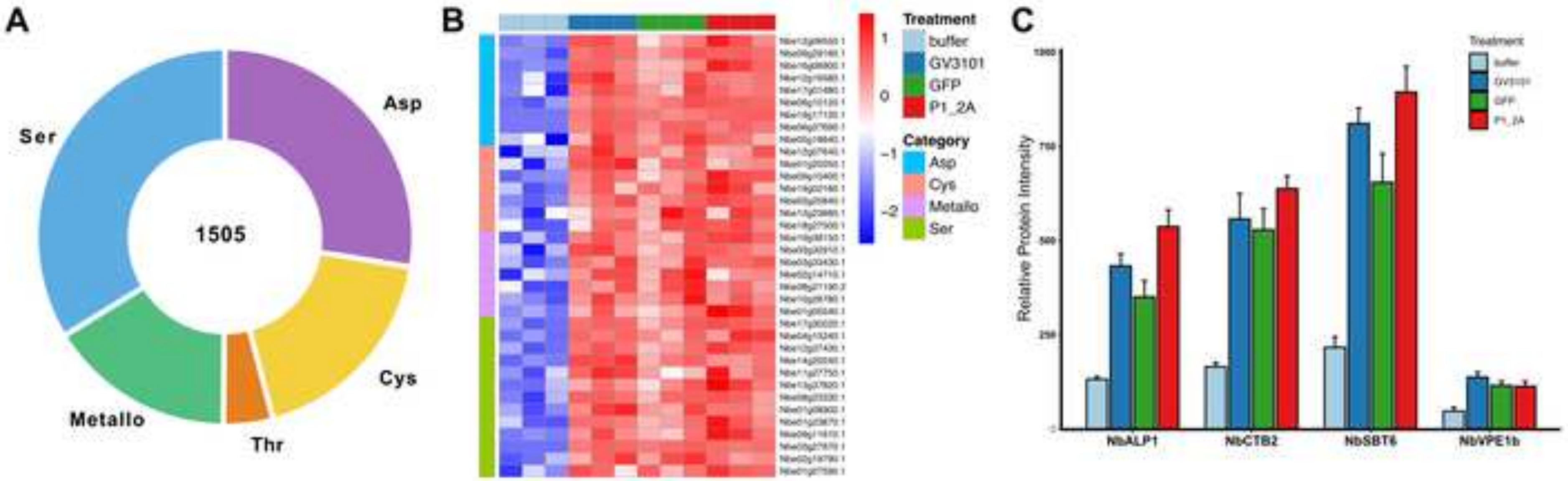

Fig 5

Click here to access/download;Figure;fig5.jpg

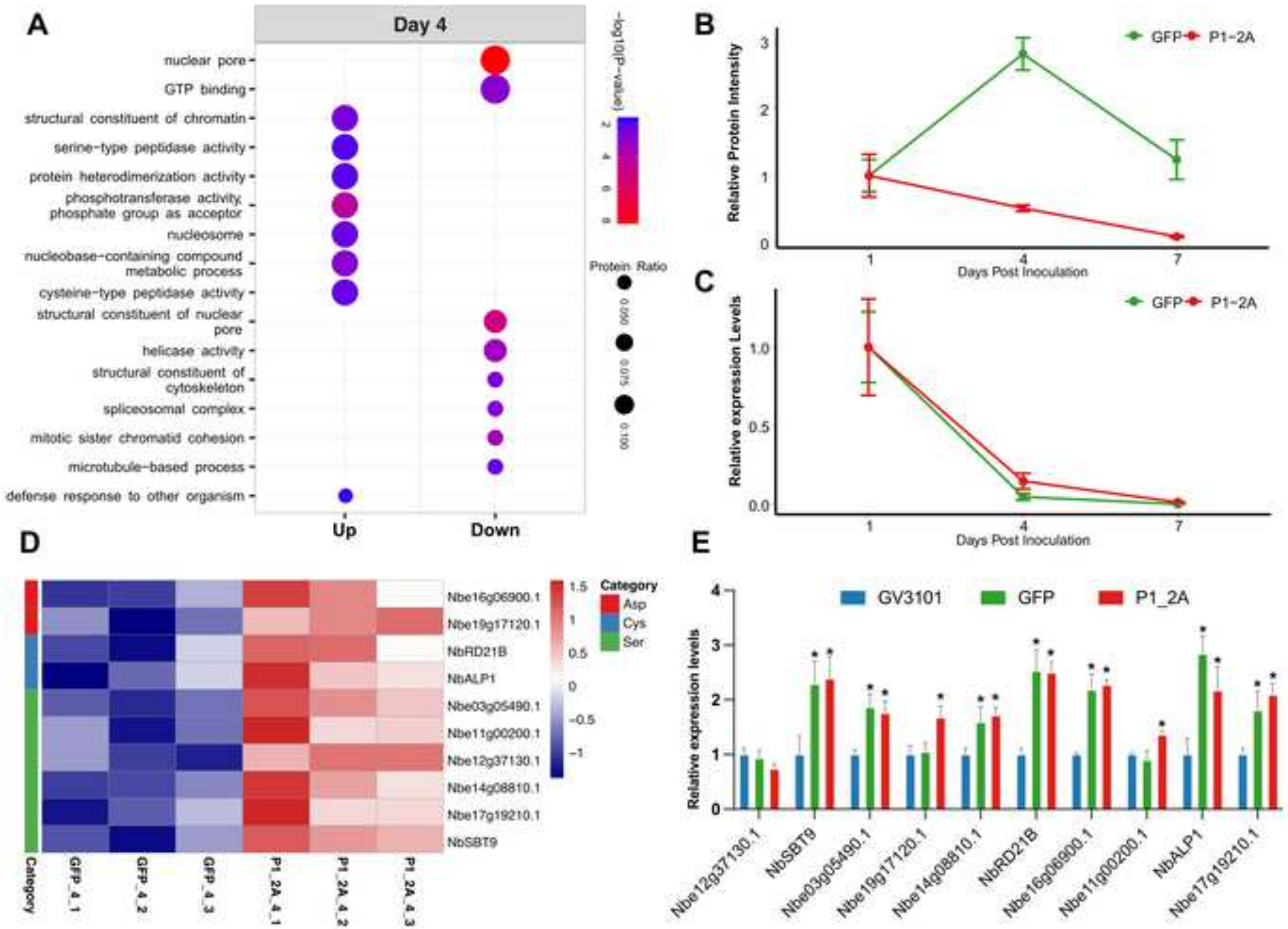

Click here to access/download

Supporting Information - Compressed/ZIP File Archive

Supplementary File.zip
