# Supplementary material for: 4D-DIA proteomics reveals distinct proteolytic landscapes induced by mechanical stress, Agrobacterium, and a viral capsid precursor
Source: PLoS One. 2026 Sep 11;21(9):e0356905. doi: 10.1371/journal.pone.0356905 (PMC13567786; doi:10.1371/journal.pone.0356905)
Supplement: Supplementary file 5 [file pone.0356905.s008.docx]

The modified version of the manuscript entitled « 4D-DIA Proteomics Reveals Distinct Proteolytic Landscapes Induced by Mechanical Stress, Agrobacterium, and Complex Viral Particles”, and submitted by Tang et al. for publication in PLOS One, is much improved compared to the first version of the manuscript. Among the improvements, I note important changes made in responses to reviewers comments, particularly with respect to the interpretation of the results, making the manuscript more rigorous scientifically. The manuscript also reads better with the changes made to the content of the results vs discussion sections. The English is also improved. I recommend the publication of this manuscript with two suggestions of modification.

- In the Author summary section, I suggest to replace “affordable” by “cost-efficient” leading to the sentence “Our findings provide a roadmap for designing product-specific engineering strategies, ultimately making plant-based medicine production more practical and cost-efficient”.
- I the results section, lines 133-135 state “Notably, the buffer-infiltrated group showed clear separation from the non-infiltrated WT group as early as 1 dpi (Fig 1D).” while Figure 1D highlights the grouping of WT and buffer treatments at 1 and 4 dpi. Figure 1D should highlight the separation of WT and buffer for 1 dpi and 4 DPI.
