# Supplementary material for: 4D-DIA proteomics reveals distinct proteolytic landscapes induced by mechanical stress, Agrobacterium, and a viral capsid precursor
Source: PLoS One. 2026 Sep 11;21(9):e0356905. doi: 10.1371/journal.pone.0356905 (PMC13567786; doi:10.1371/journal.pone.0356905)
Supplement: Supplementary file 6 [file pone.0356905.s011.docx]

# Response to Reviewers — PONE-D-26-17969 (Second Revision)

**Manuscript:** 4D-DIA proteomics reveals distinct proteolytic landscapes induced by mechanical stress, *Agrobacterium*, and a viral capsid precursor

**Notes for the editorial office:** - The **manuscript title has been changed** in this revision, at Reviewer 2’s suggestion.

We thank both Reviewers for their continued attention to this manuscript. Reviewer 1 recommended publication with two suggestions, both of which we have adopted. Reviewer 2 returned an annotated manuscript containing extensive language-level edits, which we have **accepted in full**, together with eleven substantive comments answered individually below.

Reviewer 2’s comments converged on a single issue that we had not stated carefully enough: whether the FMDV P1_2A capsid precursor actually assembled into virus-like particles in our material. We did not test this and on re-reading we found that the manuscript nevertheless implied that particles had formed. We have therefore removed every claim of particle assembly and describe the cargo as what we in fact expressed: a large, multidomain viral capsid precursor. This does not weaken the study’s conclusion, which concerns how the host proteolytic response differs between two recombinant cargoes and does not depend on whether particles assembled.

In preparing this revision we also identified several errors of our own that neither reviewer raised. All changes are marked in the tracked-changes manuscript. Point-by-point responses follow.

## Response to Reviewer 1

We thank Reviewer 1 for the positive assessment and for two precise suggestions, both of which we have adopted.

**Comment 1.1 (Author summary):** *“I suggest to replace ‘affordable’ by ‘cost-efficient’ leading to the sentence ‘Our findings provide a roadmap for designing product-specific engineering strategies, ultimately making plant-based medicine production more practical and cost-efficient’.”*

**Response:** We have adopted the reviewer’s term: *“These findings support the design of product-specific engineering strategies for more practical and* ***cost-efficient*** *plant-based medicine production.”*

**Comment 1.2 (Results, lines 133–135; Fig 1D):** *“…state ‘Notably, the buffer-infiltrated group showed clear separation from the non-infiltrated WT group as early as 1 dpi (Fig 1D).’ while Figure 1D highlights the grouping of WT and buffer treatments at 1 and 4 dpi. Figure 1D should highlight the separation of WT and buffer for 1 dpi and 4 DPI.”*

**Response:** We have rewritten the description of this panel in the revised manuscript.

The passage now describes what the panel shows: *“Per-time-point PCA (Fig 1D) quantified the same contrast within each time point separately. The agroinfiltrated and non-infiltrated groups were separated mainly along PC1, which accounted for 30.6%, 38.9% and 31.3% of the total variance at 1, 4 and 7 dpi. Agroinfiltration is therefore the largest single source of proteome variation at each time point.”*

**Changes:** Results, Fig 1D passage and legend have been rewritten.

## Response to Reviewer 2

We thank Reviewer 2 for annotating the manuscript directly, which made every point unambiguous, and for pressing on the question of what our cargo actually was. That question exposed a over-statement, and correcting it has made the manuscript more accurate.

### Title and Abstract

**Comment 2.1 (Title, on “complex”):** *“This word in the title creates some questions. If not needed to remove. You may want to say viral like particles.”*

**Response:** The reviewer is correct, and we have gone slightly further than suggested. Because we did not verify particle assembly (comments 1.6 and 1.8), the phrase “viral particles” was itself unsupported. The title now names the molecule we expressed: *“…induced by mechanical stress, Agrobacterium, and* ***a viral capsid precursor****.”*

**Changes:** Title revised.

**Comment 2.2 (Abstract, on “in plants or N. benthamiana”):** *“authors to decide.”*

**Response:** We have kept “in plants” and removed “or *N. benthamiana*”, which was redundant since *N. benthamiana* is itself a plant.

**Changes:** Abstract, final sentence.

### Author summary

**Comment 1.3 (on “complex viral antigens”):** *“to explain complexity.”*

**Response:** We agree that the adjective carried no information. It is replaced by the properties that matter: the cargo is now described as *“a large, multidomain viral capsid precursor,”* with its size and architecture given explicitly in the Methods (comment 2.7) and discussed as a possible cause of the observed protease difference (comment 2.11).

**Changes:** Author summary; Materials and Methods; Discussion.

**Comment 2.4 (on “This cargo-dependent difference suggests that protection strategies may need to be tailored to the specific product rather than applied uniformly”):** *“this is vague could you simplify it?”*

**Response:** Simplified to: *“Protection strategies may therefore need to match the specific product.”*

**Changes:** Author summary.

### Introduction

**Comment 2.5 (on “virus-like particles”):** *“this is a protein-based structure. Do authors mean that VLPs or the monomer are secreted as structures? to clarify.”*

**Response:** We meant the monomeric subunits, and the text was ambiguous. We have made the distinction explicit: *“For such cargoes, the structural proteins transit the ER and Golgi, where they undergo folding, disulfide bond formation and glycosylation before or concomitant with higher-order assembly. In most enveloped VLP systems, intracellular trafficking occurs primarily at the level of individual structural proteins or small oligomers, with particle assembly coupled to membrane localization and budding. ”*

**Changes:** Introduction, paragraph on the physicochemical properties of the recombinant product.

**Comment 2.6 (on “P1_2A”):** *“Is this producing a VLP structure? To mention capsid precursor and a reference showing the generation of VLPs.”*

**Response:** This comment identified the weakness of the previous version, and we are grateful for it. We do not know whether P1_2A assembled into particles in *N. benthamiana* in this manuscript, because we did not test it; P1_2A also accumulated to low levels throughout the time course (Fig 5B). We have accordingly (i) named the cargo a **capsid precursor** in the title, Abstract, Author summary, Introduction, Results, Discussion and Conclusions; and (ii) cited Xiao et al. [11], which reports the generation of FMDV serotype O virus-like particles, as a *reported property of the sequence* rather than an observation of ours: *“P1_2A, an animal viral capsid precursor* ***reported to*** *self-assemble into VLPs [11].”*

**Changes:** Title; Abstract; Author summary; Introduction; Results; Discussion; Conclusions.

### Materials and Methods

**Comment 1.7 (on “GFP and the FMDV P1_2A capsid precursor”):** *“to provide the size of the gene and the protein product.”*

**Response:** Both are now given: *“The coding sequences for GFP (717 bp, encoding a 27 kDa protein) and the FMDV P1_2A capsid precursor (2,340 bp, encoding an 85 kDa polyprotein)…”*

**Changes:** Materials and Methods, “Plant material and agroinfiltration”.

### Results

**Comment 2.8 (on “a significantly higher number of DEPs”):** *“Is this due to the monomer or the VLP? Any speculations? Propose resolution in Discussion.”*

**Response:** We cannot distinguish these possibilities with the present data, and we now say so and propose how it could be settled: *“Our data cannot say whether the P1_2A-specific protease response comes from the unassembled precursor, from assembled particles, or from degradation intermediates of either. Because P1_2A accumulated poorly, unassembled and partially folded precursor is the more likely source. Distinguishing these would require fractionating infiltrated tissue by density gradient or imaging it by electron microscopy, or comparing P1_2A with an assembly-deficient variant that accumulates precursor but forms no particles.”*

**Changes:** Results reworded; three sentences added to the Discussion.

### Discussion

**Comment 2.9 (on “accession”):** *“Are there other accessions used in ag science? Actually publications never refer to a specific N ben accession …”*

**Response:** All experiments used the laboratory accession **RA4**, maintained in our laboratory, which is now stated in the Methods. On whether accession identity matters, the evidence is already in our reference list: Drapal et al. [7] compared agroinfiltration responses across *N. benthamiana* accessions and reported accession-dependent metabolic differences. The limitation now reads: *“Agroinfiltration responses differ between accessions [7], so these results may not transfer to other accessions or to field-grown plants.”*

**Changes:** Materials and Methods (accession named); Discussion, Limitations.

**Comment 2.10 (on “subsequently”, T-DNA paragraph):** *“maybe further? Authors could estimate the lifespan of the T-DNA in planta as a proxy of the time the source is present.”*

**Response:** We thank the reviewer for this suggestion. We have added those sentences: *“In the binary T-DNA system used here the transgene is expressed from T-DNA that is delivered in a single wave of infection and is predominantly maintained as non-integrated episomal DNA during transient expression [27]. Neither the DNA template nor the transcript is autonomous amplified in planta, so the available pool of transcriptionally competent T-DNA is established during delivery and subsequently decreases through dilution, degradation, chromatinization, or transcriptional silencing. Product accumulation is expected to rise to an early maximum and then fall as that pool is depleted and the infiltrated tissue mounts its defense response.”*

On the estimate itself, we found no direct measurement of T-DNA lifespan in planta, and have therefore given the bracket the literature does supply while stating explicitly what it does not establish: *“Extrachromosomal T-DNA can still be recovered from agroinfiltrated leaves at 4–8 dpi [28], so the template is physically present across the window we sampled, giving a persistence on the order of a week. Whether it remains transcriptionally active throughout that period has not been established, so the 4 dpi maximum and near-baseline 7 dpi values we report cannot be attributed to template loss alone. ”*

**Changes:** Discussion, T-DNA kinetics paragraph; two references added ([27] Lacroix & Citovsky 2013; [28] Singer et al. 2012).

**Comment 2.11 (on “GFP and the FMDV P1_2A capsid precursor”):** *“the size of the protein product is not discussed as a potential factor for the cargo size differences.”*

**Response:** We agree, and this addition strengthens the Discussion by supplying a concrete physical mechanism for a difference we had described only phenomenologically: *“The two cargoes also differ in size and architecture. GFP is a 27 kDa single-domain protein that folds on its own; P1_2A is an 85 kDa polyprotein of four capsid domains that must be cleaved at the 2A site before assembly. The larger cargo folds more slowly and is processed during maturation, so it stays exposed to proteases for longer. That alone could account for part of the cargo-dependent difference, without invoking the particle.”*

**Changes:** Discussion, cargo-dependence paragraph.

## Additional Corrections Identified by the Authors

These were not raised by either reviewer. The first affects how the results should be read, and we would rather state it plainly.

**A1 — The Methods misstated our differential-expression criteria.** The previous Methods stated that DEPs were defined by *“an absolute fold change > 1.5 ”.*  This did not describe the analysis that produced our data. Table S2, and every figure and count derived from it, was generated with **a fold change > 1.2**. The analysis itself has not been changed in this revision.

**Changes:** Materials and Methods, “Bioinformatics analysis”, Fig2 legend.

**A2 — A proteomic result was described as transcriptomic.** The Discussion stated that infiltration alone was *“sufficient to prime the defense transcriptome.”* This is a proteomic study and we have no transcript-level comparison between the buffer and WT groups. Corrected to *“sufficient to induce defense-associated proteins.”*

**Changes:** Discussion.

**A3 — Supporting-information caption was too narrow.** The S2 Table caption described the table as covering the Buffer-versus-WT comparison only; it in fact contains all pairwise group comparisons.

**Changes:** S2 Table caption corrected.

**A4 — A reference was cited by the wrong number.** In the Results, the *N. benthamiana* genome assembly was cited as [15] (Kourelis et al., a proteome resource) where [14] (Wang et al., the genome assembly) was intended, as correctly cited in the Methods. **Changes:** Corrected to [14].

**A5 — References added.** Two references were added for comment 2.10. Because every previously cited work first appears earlier in the text than that paragraph, **the existing numbering [1]–[26] is unchanged**:

- **[27]** Lacroix B, Citovsky V. The roles of bacterial and host plant factors in *Agrobacterium*-mediated genetic transformation. Int J Dev Biol. 2013;57:467–81. doi:10.1387/ijdb.130199bl
- **[28]** Singer K, Shiboleth YM, Li J, Tzfira T. Formation of complex extrachromosomal T-DNA structures in *Agrobacterium tumefaciens*-infected plants. Plant Physiol. 2012;160:511–22. doi:10.1104/pp.112.200212

We believe these revisions address the points raised, and we thank both Reviewers again for their time and expertise.
