# Supplementary material for: 4D-DIA proteomics reveals distinct proteolytic landscapes induced by mechanical stress, Agrobacterium, and a viral capsid precursor
Source: PLoS One. 2026 Sep 11;21(9):e0356905. doi: 10.1371/journal.pone.0356905 (PMC13567786; doi:10.1371/journal.pone.0356905)
Supplement: Peer review history

# Peer review: Decision Letter 0

**DOI:** 10.1371/journal.pone.0356905.r001

**Authors:** Vitaly Citovsky

19 May 2026

Dear Dr. Yiming,

Thank you for submitting your manuscript to PLOS ONE. After careful consideration, we feel that it has merit but does not fully meet PLOS ONE’s publication criteria as it currently stands. Therefore, we invite you to submit a revised version of the manuscript that addresses the points raised during the review process.

Please submit your revised manuscript by Jul 03 2026 11:59PM. If you will need more time than this to complete your revisions, please reply to this message or contact the journal office at plosone@plos.org. When you're ready to submit your revision, log on to https://www.editorialmanager.com/pone/ and select the 'Submissions Needing Revision' folder to locate your manuscript file.

- A letter that responds to each point raised by the academic editor and reviewer(s). You should upload this letter as a separate file labeled 'Response to Reviewers'.
- A marked-up copy of your manuscript that highlights changes made to the original version. You should upload this as a separate file labeled 'Revised Manuscript with Track Changes'.
- An unmarked version of your revised paper without tracked changes. You should upload this as a separate file labeled 'Manuscript'.

If you would like to make changes to your financial disclosure, please include your updated statement in your cover letter. Guidelines for resubmitting your figure files are available below the reviewer comments at the end of this letter.

If applicable, we recommend that you deposit your laboratory protocols in protocols.io to enhance the reproducibility of your results. Protocols.io assigns your protocol its own identifier (DOI) so that it can be cited independently in the future. For instructions see: https://journals.plos.org/plosone/s/submission-guidelines#loc-laboratory-protocols. Additionally, PLOS ONE offers an option for publishing peer-reviewed Lab Protocol articles, which describe protocols hosted on protocols.io. Read more information on sharing protocols at https://plos.org/protocols?utm_medium=editorial-email&utm_source=authorletters&utm_campaign=protocols.

As the corresponding author, your ORCID iD is verified in the submission system and will appear in the published article. PLOS supports the use of ORCID, and we encourage all coauthors to register for an ORCID iD and use it as well. Please encourage your coauthors to verify their ORCID iD within the submission system before final acceptance, as unverified ORCID iDs will not appear in the published article. *Only*  the individual author can complete the verification step; PLOS staff *cannot*  verify ORCID iDs on behalf of authors.

We look forward to receiving your revised manuscript.

Kind regards,

Vitaly Citovsky, Ph.D.

Academic Editor

PLOS One

**Journal Requirements:**

When submitting your revision, we need you to address these additional requirements.

1. Please ensure that your manuscript meets PLOS ONE's style requirements, including those for file naming. The PLOS ONE style templates can be found at

https://journals.plos.org/plosone/s/file?id=wjVg/PLOSOne_formatting_sample_main_body.pdf and

https://journals.plos.org/plosone/s/file?id=ba62/PLOSOne_formatting_sample_title_authors_affiliations.pdf

2. Please note that PLOS One has specific guidelines on code sharing for submissions in which author-generated code underpins the findings in the manuscript. In these cases, we expect all author-generated code to be made available without restrictions upon publication of the work. Please review our guidelines at https://journals.plos.org/plosone/s/materials-and-software-sharing#loc-sharing-code and ensure that your code is shared in a way that follows best practice and facilitates reproducibility and reuse.

3. Please note that funding information should not appear in any section or other areas of your manuscript. We will only publish funding information present in the Funding Statement section of the online submission form. Please remove any funding-related text from the manuscript.

4. In the online submission form you indicate that your data is not available for proprietary reasons and have provided a contact point for accessing this data. Please note that your current contact point is a co-author on this manuscript. According to our Data Policy, the contact point must not be an author on the manuscript and must be an institutional contact, ideally not an individual. Please revise your data statement to a non-author institutional point of contact, such as a data access or ethics committee, and send this to us via return email. Please also include contact information for the third party organization, and please include the full citation of where the data can be found.

5. If the reviewer comments include a recommendation to cite specific previously published works, please review and evaluate these publications to determine whether they are relevant and should be cited. There is no requirement to cite these works unless the editor has indicated otherwise.

**Additional Editor Comments:**

May 18, 2026

Dr. Lijun Zhu

Chongqing Key Laboratory of Scientific Utilization of Tobacco Resources

Chongqing

China

Dear Dr. Zhu:

Thank you for the opportunity to read your manuscript PONE-D-26-17969 entitled “4D-DIA proteomics reveals distinct proteolytic landscapes induced by mechanical stress, Agrobacterium, and complex viral particles”. Your paper was seen by two reviewers, both of whom raised important concerns that preclude publication of this study in its present form in PLOS ONE.

However, if you can address all the reviewers’ criticisms, I will be glad to reconsider the revised paper and send it for another round of reviews. For example, it would be important to address Reviewer 1's comments experimentally by providing data at additional time points after infiltration.

Sincerely yours,

Vitaly Citovsky

Academic Editor

PLOS ONE

[Note: HTML markup is below. Please do not edit.]

Reviewers' comments:

Reviewer's Responses to Questions

**Comments to the Author**

1. Is the manuscript technically sound, and do the data support the conclusions?

Reviewer #1: Partly

Reviewer #2: Yes

**********

2. Has the statistical analysis been performed appropriately and rigorously?

Reviewer #1: I Don't Know

Reviewer #2: Yes

**********

3. Have the authors made all data underlying the findings in their manuscript fully available?

The PLOS Data policy

Reviewer #1: No

Reviewer #2: Yes

**********

4. Is the manuscript presented in an intelligible fashion and written in standard English?

Reviewer #1: Yes

Reviewer #2: No

**********

Reviewer #1: The manuscript entitled « 4D-DIA Proteomics Reveals Distinct Proteolytic Landscapes Induced by Mechanical Stress, Agrobacterium, and Complex Viral Particles”, and submitted by Tang et al. for publication in PLOS One, presents a proteomic study of plant responses to buffer vacuum infiltration, to Agrobacterium vacuum infiltration and to expression of GP and FMDV VLPs using Agrobacterium infiltration. The study expands the current understanding of plant defense responses to protein expression and suggests approaches to control plant responses and increase recombinant protein expression. However, in its current form, the results and discussion sections suffer from limitations in the analysis of the results obtained. Following are specific comments that address the limitations of the manuscript.

Materials and methods

• Line 43: the authors refer to Xiao et al. (2021) for the description of the binary plasmids used in this study. However, the paper by Xiao et al. does not present binary plasmids for the production of GFP and FMDV P1_2A in Nicotiana benthamiana. This needs to be corrected for the proper reference or the plasmids used need top be fully described.

Results

• Lines 133 to 136: the text refers to Fig 1D which shows the distinction between agroinfiltrated and un-agroinfiltrated plants (presence or absence of Agrobacterium) but describes differences linked to the physical act of infiltration. This should be corrected or clarified.

• Lines 158-160: Results indicate that buffer infiltration induce a stress response, but they also indicate that the response diminishes with time. The authors should consider this aspect of the response in their analysis.

• Lines 186-190: I’m not convinced that the reference to Prudhomme et al. (2024) provides the right support for the observation of ER protein processing components since Prudhomme et al. studied plants expressing a secreted antibody (Trastuzumab), which can be expected to impact ER protein processing. The proteins studied here, GFP and FMDV VLPs, are not secreted. The results should be presented and compared with consideration for this difference.

• Lines 199-210: The comparison with the results presented by Grosse-Holz et al. (2018) should consider the difference between the recombinant proteins studied. Grosse-Holz et al. studied the response to 3 secreted proteins. Differences in accumulation of extracellular and intracellular proteins can be expected, given the difference in localization of proteins between this study and that of Grosse-Holz et al.

• Lines 255-258: “A similar trend was observed for relative expression levels, with both recombinant proteins approaching baseline by 7 dpi, suggesting that host defense mechanisms ultimately suppress heterologous protein accumulation regardless of cargo identity”. To me, this result is reflective of a suboptimal expression system. Several publications have shown continuous increase in GFP accumulation over a week using agroinfiltration (see Matsuda et al. (2024) Journal of Agricultural meteorology 80: 29-33 for a recent report of GFP accumulation over time).

• Lines 266-269: Please explain why P1_2A would exacerbate ER stress and activate UPR-associated degradation.

• Lines 273-278: If the majority of the tested proteases showed increased transcript when expressing a recombinant protein compared to the GV3101 empty vector, haw can these be induced as a part of a “general Agrobacterium-responsive program”?

Discussion

• The discussion should consider the fact that the recombinant proteins produced in this study are cytosolic proteins whereas the majority of other studies evaluated the response to secreted proteins.

Based on the above comments, I cannot recommend the publication of this manuscript in its current form. Major revisions are needed before I can recommend the publication of the manuscript.

Reviewer #2: The manuscript has approached an issue several labs using agroinfiltration, which is the changes Agro is imposing in the agroinfiltrated plant host.

TO BRIEFLY SPECULATE ON HOW TO IMPROVE MOLECULAR PHARMING UPON OBTAINING THESE DATA.

A question remained in my mind. How to substitute agroinfiltration?

Specific suggestions are made in the uploaded annotated manuscript

**********

what does this mean?). If published, this will include your full peer review and any attached files.

If you choose “no”, your identity will remain anonymous but your review may still be made public.

**Do you want your identity to be public for this peer review?**  For information about this choice, including consent withdrawal, please see our Privacy Policy

Reviewer #1: No

Reviewer #2: No

**********

[NOTE: If reviewer comments were submitted as an attachment file, they will be attached to this email and accessible via the submission site. Please log into your account, locate the manuscript record, and check for the action link "View Attachments". If this link does not appear, there are no attachment files.]

To ensure your figures meet our technical requirements, please review our figure guidelines: https://journals.plos.org/plosone/s/figures

You may also use PLOS’s free figure tool, NAAS, to help you prepare publication quality figures: https://journals.plos.org/plosone/s/figures#loc-tools-for-figure-preparation.

NAAS will assess whether your figures meet our technical requirements by comparing each figure against our figure specifications.

Attachment Submitted filename: PONE-D-26-17969_REVIEW.pdf

# Author comment: Author response to Decision Letter 1

**DOI:** 10.1371/journal.pone.0356905.r002

22 Jul 2026

Please refer to the attached PDF file for the figures and the complete formatted response.

Response to Reviewers — PONE-D-26-17969

Manuscript: 4D-DIA Proteomics Reveals Distinct Proteolytic Landscapes Induced by Mechanical Stress, Agrobacterium, and Complex Viral Particles

We sincerely thank the Academic Editor and both Reviewers for their careful and constructive evaluation of our manuscript. The comments have substantially improved both the rigor and the clarity of the work. In this revision we have (i) added time-course data on recombinant-protein accumulation by Western blot (two biological replicates) and by whole-plant UV fluorescence imaging; (ii) added a time-resolved qRT-PCR analysis of canonical UPR/ER-stress markers across the buffer and Agrobacterium treatment groups; (iii) deposited the raw mass spectrometry data in a public repository; (iv) corrected an erroneous reference and the description of the expression plasmids; and (v) carried out a thorough revision of writing, terminology, Results/Discussion separation, and figure quality. All changes are marked in the tracked-changes manuscript. Point-by-point responses follow.

Response to the Academic Editor

Editor comment: “It would be important to address Reviewer 1’s comments experimentally by providing data at additional time points after infiltration.”

Response: We thank the Editor for this clear guidance, which we have addressed experimentally. We added independent, orthogonal measurements of recombinant-protein accumulation over the infiltration time course:

1.Western blot (anti-GFP), two independent biological replicates, sampled at 1, 4 and 7 dpi. In both replicates GFP accumulation rose to a sharp maximum at 4 dpi and declined markedly by 7 dpi (Response Figure R1B).

2.Whole-plant GFP fluorescence under UV illumination at 1, 4 and 7 dpi, which shows the same pattern at the macroscopic scale (peak green fluorescence at 4 dpi, strongly reduced by 7 dpi; Response Figure R1A). As this is a qualitative observation under non-standardized illumination, we present it as visual corroboration in this response letter.

3.A time-resolved qRT-PCR analysis of ER-stress/UPR markers (NbBiP, NbPDI, NbbZIP60, NbCRT; see Reviewer 1, comment on lines 266–269) across the buffer, empty-vector and cargo groups at 1, 4 and 7 dpi, provided as Supplementary Figure S1 and interpreted in the revised Discussion rather than added to the main-text Results.

These results confirm that the decline in recombinant-protein abundance by 7 dpi is a reproducible feature of this binary T-DNA expression system rather than an artefact, and they are fully consistent with the MS1-based proteomic time course in Figure 5.

Changes: GFP time-course evidence (anti-GFP Western blot, two biological replicates; whole-plant UV fluorescence) is presented as Response Figure R1 for the Editor and reviewers and is not incorporated into the manuscript, so no Methods text was added for it. The UPR-marker qRT-PCR is added as Supplementary Figure S1 and interpreted in the Discussion, with its procedure described in the Materials and Methods.

Response Figure R1

Response Figure R1A — Whole-plant GFP fluorescence under UV illumination at 1, 4 and 7 dpi.

Response Figure R1B — Anti-GFP Western blot of two independent biological replicates at 1, 4 and 7 dpi (NC, non-infiltrated negative control; molecular-mass markers 70/55/35/25/15 kDa; the ~27 kDa GFP band is indicated).

Response Figure R1. GFP accumulation peaks at 4 dpi and declines by 7 dpi in this binary T-DNA system. (A) Whole-plant GFP fluorescence under UV illumination at 1, 4 and 7 dpi: green fluorescence is faint at 1 dpi, strongest at 4 dpi, and visibly reduced at 7 dpi. (B) Anti-GFP Western blot of two independent biological replicates: the ~27 kDa GFP band is undetectable in the non-infiltrated control (NC), weak at 1 dpi, maximal at 4 dpi, and reduced at 7 dpi in both replicates. Both readouts are concordant with the MS1-based proteomic time course (Figure 5B).

Response to Journal Requirements

JR1 — Style/formatting and file naming. We have reformatted the manuscript and files to the PLOS ONE templates. Changes: Title page, headings, and file names conform to the PLOS ONE samples.

JR2 — Code sharing. No custom or bespoke code was used in this study. All analyses were carried out with standard, publicly available software using documented parameters — Spectronaut (v20, Biognosys) for DIA spectral processing, and HMMER (v3.3) with the MEROPS/Pfam databases for protease-domain classification — and the procedures are described in sufficient detail in the Materials and Methods to be reproduced. Changes: The Methods specify the software, versions and parameters used; no separate code repository is required.

JR3 — Funding information removed from the manuscript. Changes: The “Funding” sentence has been removed from the manuscript body; funding information is provided only in the online submission Funding Statement.

JR4 — Data availability / non-author contact. To fully comply with the PLOS Data Policy, we have deposited the raw mass spectrometry proteomics data in a public repository (ProteomeXchange, accession PXD080161, via the iProX partner repository). A non-author contact point is therefore no longer required. Changes: The Data Availability statement now reads: “The mass spectrometry proteomics data have been deposited to the ProteomeXchange Consortium via the iProX partner repository with the dataset identifier PXD080161. The dataset is private during peer review and will be made publicly available on publication; reviewer access can be arranged through the editorial office on request. All other relevant data are within the manuscript and its Supporting Information files.” (This also addresses Reviewer 1’s data-availability concern, Q3.)

JR5 — Evaluation of recommended/cited works. We reviewed the cited literature for relevance and accuracy and, in doing so, corrected a bibliographic error (see Reviewer 1, lines 186–190 and our Reference correction below).

Response to Reviewer 1

We thank Reviewer 1 for the detailed and expert reading of our manuscript. The comments identified two genuine weaknesses — an over-interpretation of the cargo-specific stress response, and insufficient attention to the secreted-versus-cytosolic nature of the recombinant proteins — that we have now addressed with new data and a more careful analysis.

Materials and Methods

Comment 1.1 (Line 43): Xiao et al. (2021) does not present binary plasmids for GFP and FMDV P1_2A in N. benthamiana; this needs to be corrected or the plasmids fully described.

Response: The reviewer is correct, and we apologize for the imprecise citation. Xiao et al. (2021) was the source of the FMDV serotype O P1_2A coding sequence only; the expression constructs themselves were generated in-house. The P1_2A coding sequence, a codon-optimized sequence taken from Xiao et al. (2021) (we did not perform the optimization ourselves), was chemically synthesized and cloned into the pEAQ-HT binary vector; the GFP control was cloned into the same backbone; the empty pEAQ-HT vector served as the vector control. We have rewritten the Methods to describe the constructs explicitly and to cite Xiao et al. (2021) appropriately as the sequence source.

Changes: Materials and Methods, “Plant materials and agroinfiltration” revised accordingly.

Results

Comment 1.2 (Lines 133–136): The text refers to Fig 1D (presence/absence of Agrobacterium) but describes differences linked to the physical act of infiltration.

Response: We thank the reviewer for catching this inconsistency. The separation highlighted in Fig 1D is between buffer-infiltrated and untreated (WT) tissue — i.e., the effect of the physical infiltration event — not the presence/absence of Agrobacterium. We have corrected the text so that the description matches the comparison shown.

Changes: Results reworded to refer to the buffer-vs-WT (mechanical) comparison.

Comment 1.3 (Lines 158–160): Buffer infiltration induces a stress response that diminishes with time; the authors should consider this in their analysis.

Response: We agree and have incorporated this explicitly. We now note that the buffer-induced response is strongest early and attenuates over the 1–7 dpi window, and we discuss this temporal decay when interpreting the “background” against which the Agrobacterium-specific response is defined.

Changes: Results and Discussion updated to address the time-dependence of the mechanical-stress response.

Comment 1.4 (Lines 186–190): Prudhomme et al. (2024) studied a secreted antibody (Trastuzumab), which is expected to affect ER protein processing; GFP and FMDV VLPs are not secreted. Results should be presented with this difference in mind.

Response: We have revised the text accordingly. We now make clear that Prudhomme et al. (2024) examined a secreted glycoprotein, whereas the cargoes in our study (GFP and FMDV P1_2A) are cytosolic and do not transit the secretory pathway. Because Reviewer 2 also asked that interpretive comparisons be kept out of the Results (comment 2.1), we have moved this point to the Discussion: a dedicated Discussion paragraph now explains the secreted-versus-cytosolic distinction and explicitly cautions against extrapolating secreted-protein ER effects to our cytosolic cargoes, while the Results simply report the observed upregulation of ER protein-processing components.

We also note that, in correcting our citations, we found that the reference we had used for transient UPR induction was bibliographically incorrect: the study by Hamel et al. on influenza haemagglutinin-induced UPR is published in Plant Biotechnology Journal (2024) 22:1146–1163, doi:10.1111/pbi.14252, not in the journal/volume previously cited. This has been corrected.

Changes: Prudhomme ER-processing comparison removed from the Results and the secreted-vs-cytosolic caveat relocated to a dedicated Discussion paragraph (see Reviewer 2, 2.1); Reference list corrected (Hamel et al. 2024).

Comment 1.5 (Lines 199–210): The comparison with Grosse-Holz et al. (2018) should consider that they studied three secreted proteins; extracellular vs intracellular accumulation may differ.

Response: We agree. We have added the caveat that Grosse-Holz et al. (2018) characterized the response to secreted recombinant proteins and analyzed the extracellular/secretome compartment, whereas our total-proteome analysis of cytosolic cargoes captures a different protein population. We now frame our comparison around this distinction rather than treating the two settings as equivalent.

Changes: Results and Discussion revised.

Comment 1.6 (Lines 255–258): “…both recombinant proteins approaching baseline by 7 dpi… regardless of cargo identity.” This is reflective of a suboptimal expression system; several publications show continuous GFP accumulation over a week (e.g., Matsuda et al. 2024).

Response: We thank the reviewer for this important comment, which prompted the additional time-course experiments requested by the Editor. We respectfully suggest that the decline we observe is not evidence of a suboptimal system but a genuine and reproducible property of the binary T-DNA platform we used, and that the apparent discrepancy with reports of sustained GFP accumulation reflects a difference in expression system:

•Our new Western blots (two biological replicates) and whole-plant UV fluorescence imaging independently confirm that GFP accumulation peaks at 4 dpi and declines by 7 dpi, in agreement with the MS1-based proteomic time course (Response Figure R1).

•Matsuda et al. (2024) used a viral replicon (tobamovirus) system, in which continuous replication of the transcript sustains and amplifies reporter accumulation, whereas we used a binary T-DNA vector, which is transcribed once and is not amplified. In such non-amplified agroinfiltration systems, an early peak at ~4–5 dpi followed by decline is well documented: (i) in a pEAQ-based reporter-optimization study in N. benthamiana, GUS activity peaked at 4 dpi (strains AGL1 and LBA4404) and declined to undetectable levels by 8 dpi (Norkunas et al. 2018); (ii) a pEAQ-expressed pharmaceutical protein (GA733-Fc) reached maximal accumulation at 5 dpi and decreased thereafter (Kim et al. 2021); and (iii) transiently expressed SARS-CoV-2 RBD was highest at 4 dpi and then gradually decreased (Maharjan et al. 2021). The decline we observe by 7 dpi is therefore the expected behaviour of a non-amplified binary T-DNA platform, not a sign of a suboptimal system.

•The two systems are not actually in conflict over the relevant window: Matsuda et al. (2024) themselves report a plateau at ~6–8 dpi, overlapping our 4–7 dpi window; the difference is that ongoing viral replication in their system masks the host-driven decline, which our non-amplified binary T-DNA system reveals. The vacuum infiltration used here additionally imposes stronger mechanical stress than the syringe infiltration of most cited studies, which would further accentuate the early host response.

We have rewritten this passage to (i) present the new orthogonal data, (ii) attribute the kinetics explicitly to the binary T-DNA system, and (iii) remove any implication of a universal “suppression regardless of cargo.”

Changes: Results rewritten; GFP time-course evidence presented as Response Figure R1 (Western blot + UV fluorescence); Discussion contrasts binary T-DNA vs viral-replicon kinetics.

Comment 1.7 (Lines 266–269): Please explain why P1_2A would exacerbate ER stress and activate UPR-associated degradation.

Response: This comment identified an over-interpretation on our part, which we have now tested directly and corrected. We performed time-resolved qRT-PCR of canonical UPR/ER-stress markers (NbBiP, NbPDI, NbbZIP60, NbCRT) across the buffer, empty-vector (GV3101) and cargo (GFP, P1_2A) groups at 1, 4 and 7 dpi (n = 3). The results show that:

•buffer infiltration does not induce these markers (≈1× throughout);

•all Agrobacterium-treated groups induce them strongly (≈4–22× over buffer); and

•the magnitude of UPR induction tracks recombinant-protein accumulation rather than cargo “complexity.” GFP — the high-accumulating cargo — showed the strongest UPR (e.g., NbBiP ≈22× at 4 dpi, coincident with its protein peak), whereas P1_2A, which accumulates poorly, showed the weakest UPR of the three Agrobacterium groups (NbBiP ≈4× at 4 dpi).

Thus our own data do not support a P1_2A-specific exacerbation of ER stress, and we have removed that claim. We also note a mechanistic rationale consistent with the reviewer’s broader concern: the reports of VLP/glycoprotein-induced UPR (Hamel et al. 2024, 2025) concern ER-client secretory glycoproteins (influenza haemagglutinin; coronavirus spike), whereas GFP and FMDV P1_2A are cytosolic, so a cargo-specific UPR is not expected. Accordingly, we now interpret the cargo-associated differences in the protease proteome as decoupled from the UPR, and we state that their mechanistic basis remains to be established.

Changes: Removed the ER-stress/UPR mechanistic claim (former lines 266–269); the two references previously cited for that claim (Hatsugai et al. and Thomas) are retained only as a neutral attribution of the vacuolar processing enzymes (VPEs) detected in the protease signature, and are no longer cited in support of a UPR-driven degradation mechanism. We added the UPR-marker qRT-PCR as Supplementary Figure S1 and describe it in the Discussion (not the main-text Results), reframing UPR as an accumulation-linked, Agrobacterium-general response and the cargo-specific protease differences as an observation whose mechanism is not yet resolved.

Supplementary Figure S1. UPR/ER-stress marker transcripts during the infiltration time course. Relative expression of NbBiP, NbPDI, NbbZIP60 and NbCRT (qRT-PCR; n = 3) in Buffer, GV3101 (empty vector), GFP and P1_2A tissues at 1, 4 and 7 dpi. Buffer remains at ≈1 throughout (mechanical infiltration does not induce the UPR); all Agrobacterium groups are strongly induced; induction tracks recombinant-protein accumulation, peaking in GFP at 4 dpi (e.g., NbBiP ≈22×) and remaining lowest in the poorly accumulating P1_2A group.

Comment 1.8 (Lines 273–278): If most tested proteases showed increased transcript when expressing a recomb

Attachment Submitted filename: Response_to_Reviewers_PONE-D-26-17969.pdf

# Peer review: Decision Letter 1

**DOI:** 10.1371/journal.pone.0356905.r003

**Authors:** Vitaly Citovsky

3 Aug 2026

Dear Dr. Sun,

Thank you for submitting your manuscript to PLOS ONE. After careful consideration, we feel that it has merit but does not fully meet PLOS ONE’s publication criteria as it currently stands. Therefore, we invite you to submit a revised version of the manuscript that addresses the points raised during the review process.

==============================

Dear Dr. Zhu:

Thank you for the opportunity to read your revised manuscript PONE-D-26-17969R1 entitled “4D-DIA proteomics reveals distinct proteolytic landscapes induced by mechanical stress, Agrobacterium, and complex viral particles”. Your paper was seen by both original reviewers, both of whom recommended publication after relatively minor revisions.

Sincerely yours,

Vitaly Citovsky

Academic Editor

PLOS ONE

For Lab, Study and Registered Report Protocols: These article types are not expected to include results but may include pilot data.

==============================

Please submit your revised manuscript by Sep 17 2026 11:59PM. If you will need more time than this to complete your revisions, please reply to this message or contact the journal office at plosone@plos.org. When you're ready to submit your revision, log on to https://www.editorialmanager.com/pone/ and select the 'Submissions Needing Revision' folder to locate your manuscript file.

- A letter that responds to each point raised by the academic editor and reviewer(s). You should upload this letter as a separate file labeled 'Response to Reviewers'.
- A marked-up copy of your manuscript that highlights changes made to the original version. You should upload this as a separate file labeled 'Revised Manuscript with Track Changes'.
- An unmarked version of your revised paper without tracked changes. You should upload this as a separate file labeled 'Manuscript'.

If you would like to make changes to your financial disclosure, please include your updated statement in your cover letter. Guidelines for resubmitting your figure files are available below the reviewer comments at the end of this letter.

If applicable, we recommend that you deposit your laboratory protocols in protocols.io to enhance the reproducibility of your results. Protocols.io assigns your protocol its own identifier (DOI) so that it can be cited independently in the future. For instructions see: https://journals.plos.org/plosone/s/submission-guidelines#loc-laboratory-protocols. Additionally, PLOS ONE offers an option for publishing peer-reviewed Lab Protocol articles, which describe protocols hosted on protocols.io. Read more information on sharing protocols at https://plos.org/protocols?utm_medium=editorial-email&utm_source=authorletters&utm_campaign=protocols.

As the corresponding author, your ORCID iD is verified in the submission system and will appear in the published article. PLOS supports the use of ORCID, and we encourage all coauthors to register for an ORCID iD and use it as well. Please encourage your coauthors to verify their ORCID iD within the submission system before final acceptance, as unverified ORCID iDs will not appear in the published article. *Only*  the individual author can complete the verification step; PLOS staff *cannot*  verify ORCID iDs on behalf of authors.

We look forward to receiving your revised manuscript.

Kind regards,

Vitaly Citovsky, Ph.D.

Academic Editor

PLOS One

Journal Requirements:

1. If the reviewer comments include a recommendation to cite specific previously published works, please review and evaluate these publications to determine whether they are relevant and should be cited. There is no requirement to cite these works unless the editor has indicated otherwise.

2. Please review your reference list to ensure that it is complete and correct. If you have cited papers that have been retracted, please include the rationale for doing so in the manuscript text, or remove these references and replace them with relevant current references. Any changes to the reference list should be mentioned in the rebuttal letter that accompanies your revised manuscript. If you need to cite a retracted article, indicate the article’s retracted status in the References list and also include a citation and full reference for the retraction notice.

[Note: HTML markup is below. Please do not edit.]

Reviewers' comments:

Reviewer's Responses to Questions

**Comments to the Author**

Reviewer #1: All comments have been addressed

Reviewer #2: All comments have been addressed

**********

2. Is the manuscript technically sound, and do the data support the conclusions?

Reviewer #1: Yes

Reviewer #2: Yes

**********

3. Has the statistical analysis been performed appropriately and rigorously?

Reviewer #1: I Don't Know

Reviewer #2: Yes

**********

4. Have the authors made all data underlying the findings in their manuscript fully available?

The PLOS Data policy

Reviewer #1: Yes

Reviewer #2: (No Response)

**********

5. Is the manuscript presented in an intelligible fashion and written in standard English?

Reviewer #1: Yes

Reviewer #2: Yes

**********

Reviewer #1: The modified version of the manuscript entitled « 4D-DIA Proteomics Reveals Distinct Proteolytic Landscapes Induced by Mechanical Stress, Agrobacterium, and Complex Viral Particles”, and submitted by Tang et al. for publication in PLOS One, is much improved compared to the first version of the manuscript. Among the improvements, I note important changes made in responses to reviewers comments, particularly with respect to the interpretation of the results, making the manuscript more rigorous scientifically. The manuscript also reads better with the changes made to the content of the results vs discussion sections. The English is also improved. I recommend the publication of this manuscript with two suggestions of modification.

- In the Author summary section, I suggest to replace “affordable” by “cost-efficient” leading to the sentence “Our findings provide a roadmap for designing product-specific engineering strategies, ultimately making plant-based medicine production more practical and cost-efficient”.

- I the results section, lines 133-135 state “Notably, the buffer-infiltrated group showed clear separation from the non-infiltrated WT group as early as 1 dpi (Fig 1D).” while Figure 1D highlights the grouping of WT and buffer treatments at 1 and 4 dpi. Figure 1D should highlight the separation of WT and buffer for 1 dpi and 4 DPI.

Reviewer #2: The revised manuscript is fluent to the reader.

The minor wording details are noted in the annotated version uploaded.

My question is whether in an expeiment where we agroinfiltrate with different cargo would there be an effcet on the buffer and Agrobacterium differences.

The size difference of GFP and P1_2a is not described or discussed as a potential factor for the differences seen.

The images seem of low resolution.

**********

what does this mean?). If published, this will include your full peer review and any attached files.

If you choose “no”, your identity will remain anonymous but your review may still be made public.

**Do you want your identity to be public for this peer review?**  For information about this choice, including consent withdrawal, please see our Privacy Policy

Reviewer #1: No

Reviewer #2: No

**********

[NOTE: If reviewer comments were submitted as an attachment file, they will be attached to this email and accessible via the submission site. Please log into your account, locate the manuscript record, and check for the action link "View Attachments". If this link does not appear, there are no attachment files.]

To ensure your figures meet our technical requirements, please review our figure guidelines: https://journals.plos.org/plosone/s/figures

You may also use PLOS’s free figure tool, NAAS, to help you prepare publication quality figures: https://journals.plos.org/plosone/s/figures#loc-tools-for-figure-preparation.

NAAS will assess whether your figures meet our technical requirements by comparing each figure against our figure specifications.

Attachment Submitted filename: Article review Tang et al 2026_resubmission.docx

Attachment Submitted filename: Revised_Manuscript_clean_REVIEW.docx

# Author comment: Author response to Decision Letter 2

**DOI:** 10.1371/journal.pone.0356905.r004

8 Aug 2026

Response to Reviewers — PONE-D-26-17969 (Second Revision)

Manuscript: 4D-DIA proteomics reveals distinct proteolytic landscapes induced by mechanical stress, Agrobacterium, and a viral capsid precursor

Notes for the editorial office: - The manuscript title has been changed in this revision, at Reviewer 2’s suggestion.

We thank both Reviewers for their continued attention to this manuscript. Reviewer 1 recommended publication with two suggestions, both of which we have adopted. Reviewer 2 returned an annotated manuscript containing extensive language-level edits, which we have accepted in full, together with eleven substantive comments answered individually below.

Reviewer 2’s comments converged on a single issue that we had not stated carefully enough: whether the FMDV P1_2A capsid precursor actually assembled into virus-like particles in our material. We did not test this and on re-reading we found that the manuscript nevertheless implied that particles had formed. We have therefore removed every claim of particle assembly and describe the cargo as what we in fact expressed: a large, multidomain viral capsid precursor. This does not weaken the study’s conclusion, which concerns how the host proteolytic response differs between two recombinant cargoes and does not depend on whether particles assembled.

In preparing this revision we also identified several errors of our own that neither reviewer raised. All changes are marked in the tracked-changes manuscript. Point-by-point responses follow.

Response to Reviewer 1

We thank Reviewer 1 for the positive assessment and for two precise suggestions, both of which we have adopted.

Comment 1.1 (Author summary): “I suggest to replace ‘affordable’ by ‘cost-efficient’ leading to the sentence ‘Our findings provide a roadmap for designing product-specific engineering strategies, ultimately making plant-based medicine production more practical and cost-efficient’.”

Response: We have adopted the reviewer’s term: “These findings support the design of product-specific engineering strategies for more practical and cost-efficient plant-based medicine production.”

Comment 1.2 (Results, lines 133–135; Fig 1D): “…state ‘Notably, the buffer-infiltrated group showed clear separation from the non-infiltrated WT group as early as 1 dpi (Fig 1D).’ while Figure 1D highlights the grouping of WT and buffer treatments at 1 and 4 dpi. Figure 1D should highlight the separation of WT and buffer for 1 dpi and 4 DPI.”

Response: We have rewritten the description of this panel in the revised manuscript.

The passage now describes what the panel shows: “Per-time-point PCA (Fig 1D) quantified the same contrast within each time point separately. The agroinfiltrated and non-infiltrated groups were separated mainly along PC1, which accounted for 30.6%, 38.9% and 31.3% of the total variance at 1, 4 and 7 dpi. Agroinfiltration is therefore the largest single source of proteome variation at each time point.”

Changes: Results, Fig 1D passage and legend have been rewritten.

Response to Reviewer 2

We thank Reviewer 2 for annotating the manuscript directly, which made every point unambiguous, and for pressing on the question of what our cargo actually was. That question exposed a over-statement, and correcting it has made the manuscript more accurate.

Title and Abstract

Comment 2.1 (Title, on “complex”): “This word in the title creates some questions. If not needed to remove. You may want to say viral like particles.”

Response: The reviewer is correct, and we have gone slightly further than suggested. Because we did not verify particle assembly (comments 1.6 and 1.8), the phrase “viral particles” was itself unsupported. The title now names the molecule we expressed: “…induced by mechanical stress, Agrobacterium, and a viral capsid precursor.”

Changes: Title revised.

Comment 2.2 (Abstract, on “in plants or N. benthamiana”): “authors to decide.”

Response: We have kept “in plants” and removed “or N. benthamiana”, which was redundant since N. benthamiana is itself a plant.

Changes: Abstract, final sentence.

Author summary

Comment 1.3 (on “complex viral antigens”): “to explain complexity.”

Response: We agree that the adjective carried no information. It is replaced by the properties that matter: the cargo is now described as “a large, multidomain viral capsid precursor,” with its size and architecture given explicitly in the Methods (comment 2.7) and discussed as a possible cause of the observed protease difference (comment 2.11).

Changes: Author summary; Materials and Methods; Discussion.

Comment 2.4 (on “This cargo-dependent difference suggests that protection strategies may need to be tailored to the specific product rather than applied uniformly”): “this is vague could you simplify it?”

Response: Simplified to: “Protection strategies may therefore need to match the specific product.”

Changes: Author summary.

Introduction

Comment 2.5 (on “virus-like particles”): “this is a protein-based structure. Do authors mean that VLPs or the monomer are secreted as structures? to clarify.”

Response: We meant the monomeric subunits, and the text was ambiguous. We have made the distinction explicit: “For such cargoes, the structural proteins transit the ER and Golgi, where they undergo folding, disulfide bond formation and glycosylation before or concomitant with higher-order assembly. In most enveloped VLP systems, intracellular trafficking occurs primarily at the level of individual structural proteins or small oligomers, with particle assembly coupled to membrane localization and budding. ”

Changes: Introduction, paragraph on the physicochemical properties of the recombinant product.

Comment 2.6 (on “P1_2A”): “Is this producing a VLP structure? To mention capsid precursor and a reference showing the generation of VLPs.”

Response: This comment identified the weakness of the previous version, and we are grateful for it. We do not know whether P1_2A assembled into particles in N. benthamiana in this manuscript, because we did not test it; P1_2A also accumulated to low levels throughout the time course (Fig 5B). We have accordingly (i) named the cargo a capsid precursor in the title, Abstract, Author summary, Introduction, Results, Discussion and Conclusions; and (ii) cited Xiao et al. [11], which reports the generation of FMDV serotype O virus-like particles, as a reported property of the sequence rather than an observation of ours: “P1_2A, an animal viral capsid precursor reported to self-assemble into VLPs [11].”

Changes: Title; Abstract; Author summary; Introduction; Results; Discussion; Conclusions.

Materials and Methods

Comment 1.7 (on “GFP and the FMDV P1_2A capsid precursor”): “to provide the size of the gene and the protein product.”

Response: Both are now given: “The coding sequences for GFP (717 bp, encoding a 27 kDa protein) and the FMDV P1_2A capsid precursor (2,340 bp, encoding an 85 kDa polyprotein)…”

Changes: Materials and Methods, “Plant material and agroinfiltration”.

Results

Comment 2.8 (on “a significantly higher number of DEPs”): “Is this due to the monomer or the VLP? Any speculations? Propose resolution in Discussion.”

Response: We cannot distinguish these possibilities with the present data, and we now say so and propose how it could be settled: “Our data cannot say whether the P1_2A-specific protease response comes from the unassembled precursor, from assembled particles, or from degradation intermediates of either. Because P1_2A accumulated poorly, unassembled and partially folded precursor is the more likely source. Distinguishing these would require fractionating infiltrated tissue by density gradient or imaging it by electron microscopy, or comparing P1_2A with an assembly-deficient variant that accumulates precursor but forms no particles.”

Changes: Results reworded; three sentences added to the Discussion.

Discussion

Comment 2.9 (on “accession”): “Are there other accessions used in ag science? Actually publications never refer to a specific N ben accession …”

Response: All experiments used the laboratory accession RA4, maintained in our laboratory, which is now stated in the Methods. On whether accession identity matters, the evidence is already in our reference list: Drapal et al. [7] compared agroinfiltration responses across N. benthamiana accessions and reported accession-dependent metabolic differences. The limitation now reads: “Agroinfiltration responses differ between accessions [7], so these results may not transfer to other accessions or to field-grown plants.”

Changes: Materials and Methods (accession named); Discussion, Limitations.

Comment 2.10 (on “subsequently”, T-DNA paragraph): “maybe further? Authors could estimate the lifespan of the T-DNA in planta as a proxy of the time the source is present.”

Response: We thank the reviewer for this suggestion. We have added those sentences: “In the binary T-DNA system used here the transgene is expressed from T-DNA that is delivered in a single wave of infection and is predominantly maintained as non-integrated episomal DNA during transient expression [27]. Neither the DNA template nor the transcript is autonomous amplified in planta, so the available pool of transcriptionally competent T-DNA is established during delivery and subsequently decreases through dilution, degradation, chromatinization, or transcriptional silencing. Product accumulation is expected to rise to an early maximum and then fall as that pool is depleted and the infiltrated tissue mounts its defense response.”

On the estimate itself, we found no direct measurement of T-DNA lifespan in planta, and have therefore given the bracket the literature does supply while stating explicitly what it does not establish: “Extrachromosomal T-DNA can still be recovered from agroinfiltrated leaves at 4–8 dpi [28], so the template is physically present across the window we sampled, giving a persistence on the order of a week. Whether it remains transcriptionally active throughout that period has not been established, so the 4 dpi maximum and near-baseline 7 dpi values we report cannot be attributed to template loss alone. ”

Changes: Discussion, T-DNA kinetics paragraph; two references added ([27] Lacroix & Citovsky 2013; [28] Singer et al. 2012).

Comment 2.11 (on “GFP and the FMDV P1_2A capsid precursor”): “the size of the protein product is not discussed as a potential factor for the cargo size differences.”

Response: We agree, and this addition strengthens the Discussion by supplying a concrete physical mechanism for a difference we had described only phenomenologically: “The two cargoes also differ in size and architecture. GFP is a 27 kDa single-domain protein that folds on its own; P1_2A is an 85 kDa polyprotein of four capsid domains that must be cleaved at the 2A site before assembly. The larger cargo folds more slowly and is processed during maturation, so it stays exposed to proteases for longer. That alone could account for part of the cargo-dependent difference, without invoking the particle.”

Changes: Discussion, cargo-dependence paragraph.

Additional Corrections Identified by the Authors

These were not raised by either reviewer. The first affects how the results should be read, and we would rather state it plainly.

A1 — The Methods misstated our differential-expression criteria. The previous Methods stated that DEPs were defined by “an absolute fold change > 1.5 ”. This did not describe the analysis that produced our data. Table S2, and every figure and count derived from it, was generated with a fold change > 1.2. The analysis itself has not been changed in this revision.

Changes: Materials and Methods, “Bioinformatics analysis”, Fig2 legend.

A2 — A proteomic result was described as transcriptomic. The Discussion stated that infiltration alone was “sufficient to prime the defense transcriptome.” This is a proteomic study and we have no transcript-level comparison between the buffer and WT groups. Corrected to “sufficient to induce defense-associated proteins.”

Changes: Discussion.

A3 — Supporting-information caption was too narrow. The S2 Table caption described the table as covering the Buffer-versus-WT comparison only; it in fact contains all pairwise group comparisons.

Changes: S2 Table caption corrected.

A4 — A reference was cited by the wrong number. In the Results, the N. benthamiana genome assembly was cited as [15] (Kourelis et al., a proteome resource) where [14] (Wang et al., the genome assembly) was intended, as correctly cited in the Methods. Changes: Corrected to [14].

A5 — References added. Two references were added for comment 2.10. Because every previously cited work first appears earlier in the text than that paragraph, the existing numbering [1]–[26] is unchanged:

•[27] Lacroix B, Citovsky V. The roles of bacterial and host plant factors in Agrobacterium-mediated genetic transformation. Int J Dev Biol. 2013;57:467–81. doi:10.1387/ijdb.130199bl

•[28] Singer K, Shiboleth YM, Li J, Tzfira T. Formation of complex extrachromosomal T-DNA structures in Agrobacterium tumefaciens-infected plants. Plant Physiol. 2012;160:511–22. doi:10.1104/pp.112.200212

We believe these revisions address the points raised, and we thank both Reviewers again for their time and expertise.

Attachment Submitted filename: Response_to_Reviewers_PONE-D-26-17969_R2.docx

# Editor report: Decision Letter 2

**DOI:** 10.1371/journal.pone.0356905.r005

**Authors:** Vitaly Citovsky

10 Aug 2026

4D-DIA proteomics reveals distinct proteolytic landscapes induced by mechanical stress, Agrobacterium , and a viral capsid precursor

PONE-D-26-17969R2

Dear Dr. Sun,

We’re pleased to inform you that your manuscript has been judged scientifically suitable for publication and will be formally accepted for publication once it meets all outstanding technical requirements.

Within one week, you’ll receive an e-mail detailing the required amendments. When these have been addressed, you’ll receive a formal acceptance letter and your manuscript will be scheduled for publication.

An invoice will be generated when your article is formally accepted. Please note, if your institution has a publishing partnership with PLOS and your article meets the relevant criteria, all or part of your publication costs will be covered. Please make sure your user information is up-to-date by logging into Editorial Manager at Editorial Manager® and clicking the ‘Update My Information' link at the top of the page. For questions related to billing, please contact billing support.

If your institution or institutions have a press office, please notify them about your upcoming paper to help maximize its impact. If they’ll be preparing press materials, please inform our press team as soon as possible -- no later than 48 hours after receiving the formal acceptance. Your manuscript will remain under strict press embargo until 2 pm Eastern Time on the date of publication. For more information, please contact onepress@plos.org.

Kind regards,

Vitaly Citovsky, Ph.D.

Academic Editor

PLOS One

Additional Editor Comments (optional):

Reviewers' comments:

# Editor report: Acceptance letter

**DOI:** 10.1371/journal.pone.0356905.r006

**Authors:** Vitaly Citovsky

PONE-D-26-17969R2

PLOS One

Dear Dr. Sun,

I'm pleased to inform you that your manuscript has been deemed suitable for publication in PLOS One. Congratulations! Your manuscript is now being handed over to our production team.

At this stage, our production department will prepare your paper for publication. This includes ensuring the following:

* All references, tables, and figures are properly cited

* All relevant supporting information is included in the manuscript submission,

* There are no issues that prevent the paper from being properly typeset

You will receive further instructions from the production team, including instructions on how to review your proof when it is ready. Please keep in mind that we are working through a large volume of accepted articles, so please give us a few days to review your paper and let you know the next and final steps.

Lastly, if your institution or institutions have a press office, please let them know about your upcoming paper now to help maximize its impact. If they'll be preparing press materials, please inform our press team within the next 48 hours. Your manuscript will remain under strict press embargo until 2 pm Eastern Time on the date of publication. For more information, please contact onepress@plos.org.

You will receive an invoice from PLOS for your publication fee after your manuscript has reached the completed accept phase. If you receive an email requesting payment before acceptance or for any other service, this may be a phishing scheme. Learn how to identify phishing emails and protect your accounts at https://explore.plos.org/phishing.

If we can help with anything else, please email us at customercare@plos.org.

Thank you for submitting your work to PLOS One and supporting open access.

Kind regards,

PLOS One Editorial Office Staff

on behalf of

Dr. Vitaly Citovsky

Academic Editor

PLOS One
